# Mesoscopic Modeling of a Highly-Ordered Sanidic Polymer Mesophase and Comparison With Experimental Data

**DOI:** 10.1021/acs.jpcb.1c10599

**Published:** 2022-03-15

**Authors:** Emma L. Wood, Cristina Greco, Dimitri A. Ivanov, Kurt Kremer, Kostas Ch. Daoulas

**Affiliations:** † Max Planck Institute for Polymer Research, Ackermannweg 10, 55128 Mainz, Germany; ‡ Institute for Problems of Chemical Physics, Russian Academy of Sciences, Semenov Prospect 1, 142432 Chernogolovka, Russia; § Lomonosov Moscow State University, Leninskie Gory 1, 119991 Moscow, Russia; ∥ Institut de Sciences des Matériaux de Mulhouse, CNRS UMR 7361, 15 Jean Starcky, F-68057 Mulhouse, France; ⊥ Sirius University of Science and Technology, 1 Olympic Ave, 354340, Sochi, Russia

## Abstract

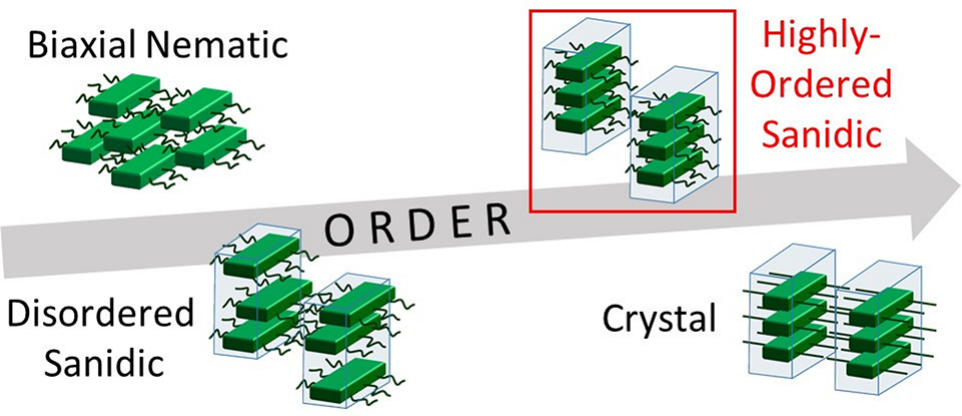

Board-shaped polymers form sanidic mesophases: assemblies of parallel lamellae of stacked polymer backbones separated by disordered side chains. Sanidics vary significantly with respect to polymer order inside their lamellae, making them “stepping stones” toward the crystalline state. Therefore, they are potentially interesting for studying crystallization and technological applications. Building on earlier mesoscopic models of the most disordered sanidics Σ_d_, we focus on the other extreme, near-crystalline order, and develop a generic model that captures a highly ordered Σ_r_ mesophase. Polymers are described by generic hindered-rotation chains. Anisotropic nonbonded potentials, with strengths comparable to the thermal energy, mimic board-like monomer shapes. Lamellae equilibrated with Monte Carlo simulations, for a broad range of model parameters, have intralamellar order typical for Σ_r_ mesophases: periodically stacked polymers that are mutually registered along their backbones. Our mesophase shows registration on both monomer and chain levels. We calculate scattering patterns and compare with data published for highly ordered sanidic mesophases of two different polymers: polyesters and polypeptoids. Most of the generic structural features that were identified in these experiments are present in our model. However, our mesophase has correlations between chains located in different lamellae and is therefore closer to the crystalline state than the experimental samples.

## 1 Introduction

**Figure 1:**
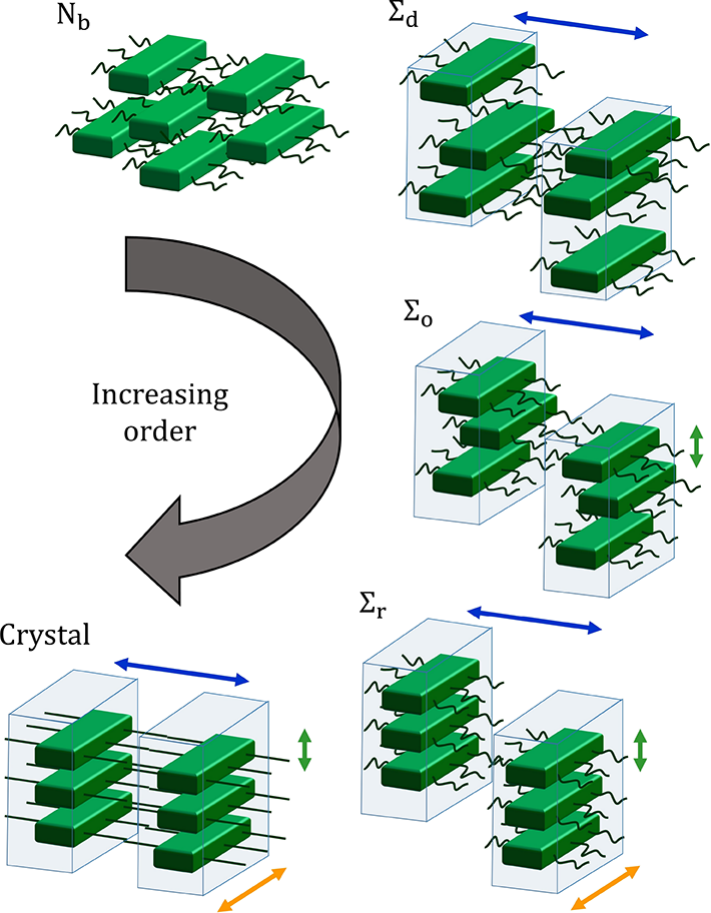
Cartoons demonstrating biaxial nematics *N*_b_, various types of sanidic Σ mesophases, and crystalline order found in materials composed of “board-shaped” polymers. These images represent polymers as rigid boards with attached disordered/ordered side chains. For simplicity, they do not show the internal degrees of freedom available to the backbones or related conformational fluctuations. Transparent blue boxes are a guide to the eye to show lamellae; boxes that are vertically shifted relative to each other indicate decorrelated lamellae. Blue arrows represent a regular lamellar spacing. Green and orange arrows represent positional order within each lamella in the stacking and backbone directions, respectively.

Many functional polymers comprise fairly large and rigid “flat” repeat units with attached side chains, rendering them “board-like” in shape.^1^ This feature affects supramolecular organization in these materials, so that their crystals present lamellae of stacked backbones alternating with layers of ordered side chains, as shown in Figure 1. Moreover, board-like molecular shapes promote ordering into mesophases^1−8^ that occupy an intermediate position between crystalline and amorphous states on the order–disorder scale. The simplest case is translationally invariant polymer biaxial nematics N_b_,^9−11^ where the two directors are oriented along and orthogonally to the backbones, respectively (see Figure 1). More intriguing, however, are sanidic mesophases, which have broken translational invariance. Here, in analogy to crystals, polymer backbones assemble into lamellae of cofacially oriented “stacks”, but now the layers of side chains that separate the lamellae are disordered.^3,6,8^

Figure 1 summarizes three basic symmetries^3^ found in sanidic mesophases: Σ_d_, Σ_o_, and Σ_r_. In “disordered sandics” Σ_d_, polymers retain two-dimensional (2D) translational freedom within each lamella, because their backbones shift arbitrarily with respect to each other, and there is no long-range positional order along the stacking direction (i.e., orthogonally to the backbones within the lamellar plane). In “ordered sanidics” Σ_o_, the chain backbones are still not registered, so may shift longitudinally, but are now regularly spaced along their stacking direction. The symmetry of the “rectangular sanidic” Σ_r_ mesophase is the closest to crystalline, exhibiting both long-range order along the stacking direction, and also registration of chain backbones. However, the positions of stacks are uncorrelated between neighboring lamellae (in contrast to crystals), at least along the stack normal.

Interest in the structure of sanidic mesophases has recently grown, partially because of organic electronics. It is argued^4−6,12^ that sanidics can serve as processing intermediates for manufacturing solid state morphologies with favorable properties, such as increased charge mobility,^5,6^ because they offer thermodynamically stable states, where board-like polymers are ordered along multiple directions. Inspecting Figure 1 reveals another interesting aspect: as new features of order emerge across the different sanidic mesophases, they increasingly reproduce generic attributes of crystals. In addition, crystalline arrangements of board-like polymers typically have substantial structural disorder,^5,13−15^ which brings them even closer to sanidic liquid crystals (LC). Hence, studying sanidic mesophases can offer (at least qualitative) insights into the properties of crystalline states in board-like polymers.

This approximation is used, often implicitly, in molecular simulations of board-like polymers on device-relevant scales. The reason is that such large-scale simulations must use computationally efficient mesoscopic models,^7,16−23^ where each effective particle represents a large number of atoms or even an entire monomer. Drastic coarse-graining smears local molecular details, which are crucial for crystallization. As a consequence, to the best of our knowledge, the various highly ordered arrangements of board-like polymers that have so far been generated with mesoscopic models are not crystals. They are partially ordered, akin to sanidic mesophases.

Nevertheless, simulations of partially ordered lamellar mesophases^7,18,20,22^ provide valuable insights. One example is the use of mesoscopic simulations^18,20^ to elucidate how free volume between side chains influences intercalation of fullerenes and, subsequently, how such intercalation affects the ordering of conjugated oligomers. These simulations were found to be in agreement with experiments^24^ on crystalline materials.

To identify universalities in structure–property relationships, it is frequently desirable for mesoscopic models to have a simple generic construction. Taking into account the relevance for approximate studies of crystalline materials, the development of generic models describing the most ordered sanidic mesophases, Σ_r_, is particularly interesting. It is challenging, however, to find a balance between simplifying the molecular description as much as possible and retaining enough detail to capture high structural order.

Here, we develop a generic model that, despite the significant reduction of chemical details, enables simulations of a Σ_r_ mesophase. We combine a drastically coarse-grained (CG) description of the molecular architecture, where each interaction site represents an entire repeat unit of a board-like polymer with special nonbonded interactions. These are expressed through potentials that are “soft” (their strength is comparable with the thermal energy *k*_B_*T*) and anisotropic. In the spirit of other models^25−33^ of LC, the choice of the anisotropicity of the interactions is “top-down”; it is guided by the symmetries of the sanidic mesophase one wants to model. Here, we build upon a strategy previously developed^7,19^ to simulate biaxial polymer nematics and the least ordered sanidics Σ_d_ and create a simple, generic model for capturing a highly ordered Σ_r_ mesophase.

We thoroughly explore the structure of our Σ_r_ mesophase and qualitatively study the phase behavior of our model across a broad range of parameter space. We compare the molecular arrangement in our mesophase with structures that have been reported in experimental studies^3,8^ of highly ordered sanidic mesophases in board-like polymers from two different families: polyesters^3^ and polypeptoids.^8^ In the experiments, the molecular organizations of the mesophases were extracted from scattering patterns. Therefore, we discuss the similarities and differences between the structure of our mesophase and the experimentally observed ones via a qualitative comparison of their scattering patterns.

## 2 Methods

To develop a mesoscopic model for Σ_r_ mesophases, we advance an approach^7,19^ that has been designed to describe less-ordered mesophases in conjugated polymers. The new model is a generic representation of board-like polymers (not necessary conjugated) and, as such, uses a minimum set of features to describe polymer architecture. Within this generic framework, we expand ideas of symmetry and interactions developed in the previous studies^7,19^ and construct a new nonbonded potential that enables modeling of a highly ordered Σ_r_ mesophase.

### 2.1 Polymer Architecture and Degrees of Freedom

Our systems contain *n* polymers described by a hindered-rotation model. Each polymer consists of *N* monomers connected by bonds with fixed length *b*.

**Figure 2:**
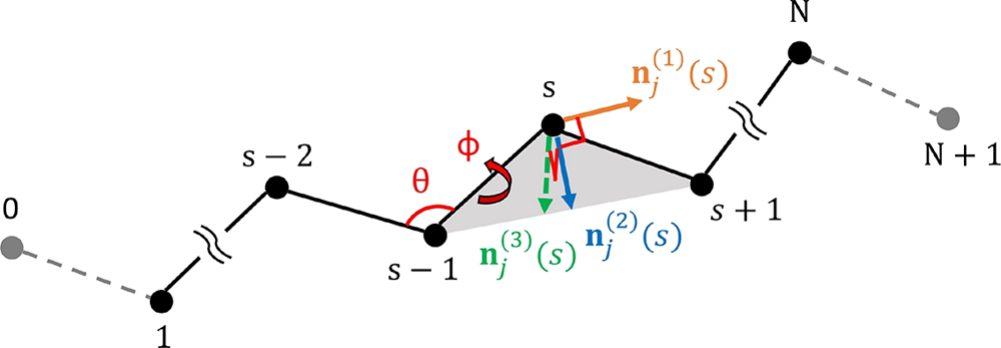
Schematic of the polymer chain architecture used in the model. Real monomers are shown as black circles, with solid black lines representing bonds of fixed length *b*. The two ghost monomers at the ends of the chains are shown in gray with dashed ghost bonds. Examples of bond and dihedral angles (θ and ϕ, respectively) are shown in red, and the orientation vectors for monomer *s* are represented by colored arrows.

The position of each CG monomer (site) in space is given by the vector **r**_*j*_(*s*), where *j* = 1, ..., *n* and *s* = 1, ..., *N* indicate the chain and the monomer, respectively. To represent an underlying anisotropic, board-like, repeat unit, each CG site also requires orientational degrees of freedom. These are introduced^7,19^ by assigning three orthonormal unit vectors {***n***_*j*_^(*k*)^(*s*)} (*k* = 1, 2, and 3) to each monomer (see Figure 2), which are fully defined by the local conformation of the chain. If ***u***_*j*_(*s*) = ***r***_*j*_(*s* + 1) – ***r***_*j*_(*s*) is a vector along the bond connecting the *s*th and (*s* + 1) th CG sites, then ***n***_*j*_^(1)^(*s*)∥***u***_*j*_(*s*) + ***u***_*j*_(*s* – 1), ***n***_*j*_^(2)^(*s*)∥***u***_*j*_(*s*) – ***u***_*j*_(*s* – 1), and ***n***_*j*_^(3)^(*s*) = ***n***_*j*_^(1)^(*s*) × ***n***_*j*_^(2)^(*s*). The orientation vectors associated with the end monomers of each chain are defined by adding ghost bonds to ghost monomers, indexed by *s* = 0 or *N* + 1, depending on which end of the polymer they are attached to. The ghost bonds are subjected to the bonded interactions of the model, but the ghost monomers do not induce any nonbonded interactions.

The bond angles θ between neighboring monomers along each polymer chain are controlled by an angular potential:
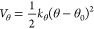
1where θ_0_ is the most probable bond angle and *k*_θ_ is the stiffness coefficient. In real materials, bond angles tend to only fluctuate a small amount around their equilibrium values,^34^ so we set *k*_θ_ to a generic high value of 100 *k*_B_*T*. Later, we find that smectic order is part of the structure of our Σ_r_ mesophase. As the most probable bond angle θ_0_ affects the local bent-shape of molecules, which is known to correlate with smectic behavior,^35^ we test the robustness of the smectic order by varying θ_0_ in a broad range from 140° to 170°.

The dihedral angles ϕ between bonds along the polymer backbone must be controlled by a torsional potential *V*_ϕ_ in order to promote board-like conformations. We require minima corresponding to the cis and trans states, at angles of ϕ = 0° and ϕ = ±180°, respectively. The final molecular shapes in a self-assembled structure are determined in combination with nonbonded potentials that favor relatively linear arrangements (see section 2.2). Therefore, there is no need to explicitly favor the trans conformation over the cis, and we consider a simple generic case, where both states are assigned a torsional potential of 0 *k*_B_*T*. Energy barriers for torsional rotation are generally low.^36^ Hence, we choose a weakly corrugated landscape for torsional rearrangements, where the maxima of the energy barriers have a generic height of 1 *k*_B_*T* and are placed at ϕ = ±90°, halfway between the minima of 0 *k*_B_*T* at ϕ = 0° and ϕ = ±180°. We use a simple form of *V*_ϕ_ that fulfills these requirements:
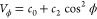
2where *c*_0_ = 1 *k*_B_*T* and *c*_2_ = −1 *k*_B_*T*.

### 2.2 Nonbonded Interactions

We define a nonbonded potential *V*_nb_ between CG monomers that either belong to different polymer chains or are separated by four or more monomers along the backbone of the same chain. Nonbonded intramolecular interactions between sites closer than four monomers apart are not activated on the grounds that such monomers are correlated by the three- and four-body bonded potentials *V*_θ_ and *V*_ϕ_.

The nonbonded potential is defined in a modular way as the sum of four different contributions, where we define β = 1/*k*_B_*T*:
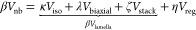
3

Each of the four components of *V*_nb_ is phenomenologically designed to promote a different feature of structural order. Their strengths can be adjusted by varying the non-negative coefficients κ, λ, ζ, and η, which are defined in units of *k*_B_*T*. We refer to the interaction defined by the sum of the first three terms of eq 3 as *V*_lamella_, the “potential for lamellar order”. This potential has been developed previously^7^ and enables the self-assembly of CG polymer chains into lamellar arrangements that reproduce the most disordered lamellar sanidic mesophase Σ_d_. In this study, we find that Σ_r_ mesophases can be formed simply by augmenting *V*_lamella_ with a generic “registration potential” *V*_reg_.

To present the model coherently and facilitate the discussion of results, we first recapitulate the main features of *V*_lamella_ (details are available elsewhere^7,19^) and then present the structure of *V*_reg_.

#### 2.2.1 Potential for Lamellar Order

Each of the three parts of *V*_lamella_ serves a specific purpose and is defined as follows:^7,19^
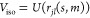
4
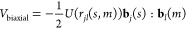
5
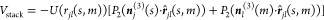
6*V*_iso_ provides finite compressibility and depends only on the distance between two particles *r*_*jl*_(*s*, *m*) = |**r**_*j*_(*s*) – **r**_*l*_(*m*)| via a repulsive isotropic core *U*(*r*) (where *r* ≡ *r*_*jl*_(*s*, *m*)). We define *U*(*r*) by^7,19^
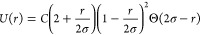
7In material-specific simulations,^7,19^ the normalization constant *C* depends on a characteristic (constant) reference density of the modeled material and is considered explicitly. In our generic simulations, there is no need to specify *C*, and we simply incorporate *C* into the definition of the coefficients κ, λ, and ζ. The range of the potential is 2σ, as indicated by the Heaviside function Θ(2σ – *r*).

Formally, eq 7 is derived^19,37^ from a generic classical density functional used in field-theory of polymers. Alternatively, the functional form of *U*(*r*) in eq 7 has a simple qualitative explanation. σ represents^7,19^ the length spanned by the side chains of the board-like monomers. Hence, in a disordered melt, each monomer has a characteristic volume *v*_0_ = 4πσ^3^/3. When two monomers approach in real materials, steric exclusions reduce the allowed conformations of side chains. Let *f*(*r*) be the free energy of a pair of monomers separated by distance *r*. When monomers are described as single objects, they interact via an effective potential that approximately equals^38^ Δ*f*(*r*) = *f*(*r*) – *f*(*r* → ∞). Simplifying even further, one can assume^38^ that Δ*f*(*r*) ∼ *v*(*r*), where *v*(*r*) is the volume of the region where the characteristic volumes *v*_0_ of the two monomers overlap. The choice of *U*(*r*) in eq 7 matches (up to a prefactor) the dependence^38^ of *v*(*r*) on *r*. To implement *V*_iso_, we therefore only need to specify σ. In all cases, we use σ = 2 *b*. In the following, all lengths will be presented in units of σ.

The dependence of *V*_biaxial_ on *r*_*jl*_(*s*, *m*) is also defined via *U*(*r*) (see eq 5). Using the same *U*(*r*) as *V*_iso_ simplifies^19^ the model and turns to be sufficient for obtaining a Σ_r_ mesophase. In addition, *V*_biaxial_ depends on the Frobenius product **b**_*j*_(*s*): **b**_*l*_(*m*) of the biaxial tensors associated with the two interacting sites. The tensor **b**_*j*_(*s*) is defined as
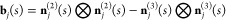
8An equivalent expression holds for **b**_*l*_(*m*). Equation 5 is a special case of a general expression^25,29,30,39^ for the quadrupolar orientational interaction energy between two biaxial particles. However, it is sufficient to define a potential with a global minimum corresponding to a relative orientation of chains where the planes of their backbones are parallel to each other, forming a biaxial nematic.

**Figure 3:**
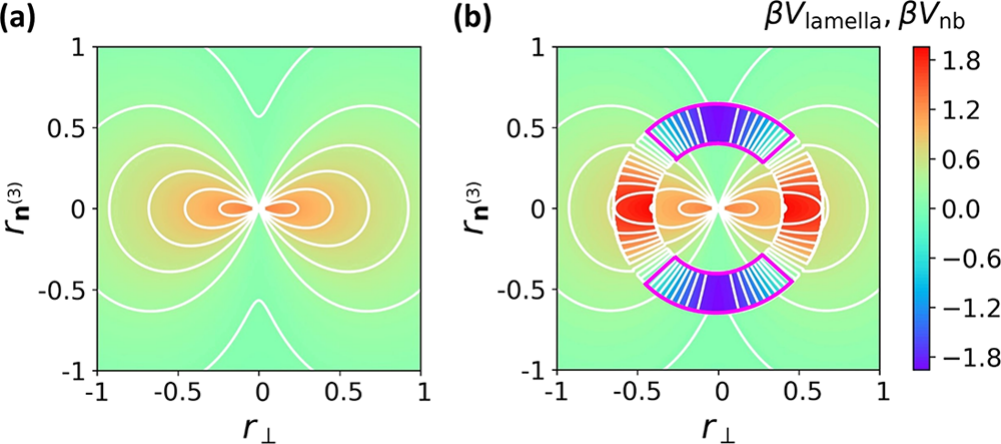
Contour plots of the nonbonded potential between two biaxially aligned monomers, as a function of the components of the intermonomer distance vector parallel (*r*_***n***^(3)^_) and perpendicular (*r*_⊥_) to the ***n***^(3)^ orientation vectors of the monomers (i.e., the stacking direction). (a) *V*_lamella_, where κ = 0.874, λ = 0.408, and ζ = 0.204 (all in units of *k*_B_*T*). (b) *V*_nb_, where κ = 0.874, λ = 0.408, ζ = 0.204, η = 1, *R*_1_ = 0.4, and *R*_2_ = 0.65. Zones where the potential is negative are outlined in magenta. In both plots, white lines highlight selected isosurfaces. The range of the potential is 2 (in units of σ), but only the central region is shown to highlight the effect of *V*_reg_.

*V*_stack_, given by eq 6, is an anisotropic “perturbation”^7^ of *V*_iso_, and is defined as convolution of *U*(*r*) with a combination of second-order Legendre polynomials *P*_2_, where **r̂**_*jl*_(*s*, *m*) = **r**_*jl*_(*s*, *m*)/*r*_*jl*_(*s*, *m*) is the unit intermonomer vector. This perturbation creates an interaction that is minimal when polymers stack face-to-face on top of each other and maximal when they are situated side by side.^7^ The combination of *V*_biaxial_ and *V*_stack_ creates an anisotropy that favors the formation of lamellae with stacks.^7^ The effect of this synergy is illustrated in Figure 3a, which presents a contour plot of *V*_lamella_ for two biaxially aligned monomers. This cylindrically symmetric plot is shown as a function of the components of the intermonomer vectors **r**_*jl*_(*s*, *m*) parallel (*r*_***n***^(3)^_) and perpendicular (*r*_⊥_) to the ***n***^(3)^ orientation vectors of the monomers (note that due to the biaxial alignment, ***n***^(3)^ is the same for both monomers). However, due to competing interactions from neighboring monomers along the chain backbones, the corrugation created by *V*_stack_ is not sufficient to break translational invariance along the backbone direction. Therefore, we augment *V*_lamella_ with a registration potential introduced in the next section.

#### 2.2.2 Registration Potential

The registration potential *V*_reg_, which provides regularity in monomer spacing along the stacking direction and prevents translational freedom along the chain backbone direction, is defined as
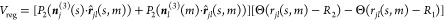
9*V*_reg_ enhances the strength of *V*_stack_ in a spherical shell between the cutoff distances *R*_1_ and *R*_2_ (where *R*_1_ < *R*_2_). This creates a zone of lower energy in *V*_nb_ (outlined in Figure 3b) that promotes the stacking of monomers at a fixed distance from each other and induces a highly ordered arrangement of monomers in the system. The cutoff *R*_2_ reduces interference between *V*_reg_ originating from different monomers along the chain, producing a stronger corrugation along the next layer of the stack than *V*_stack_. The structure of the Σ_r_ mesophase arises from a complex synergy between the potentials of all interacting particles and is also affected by density. Therefore, we cannot provide precise guidelines for choosing the cutoff distances in *V*_reg_. To reduce the parameter space, we assume *R*_1_ = *R*_2_ – 0.25 and use exploratory simulations to find suitable *R*_2_. These simulations are simplified by qualitative arguments that reduce the range of *R*_2_ values that need to be scanned (see section 3.1). In this way, we converge to the choice *R*_2_ = 0.65.

We emphasize that, for η high enough to drive Σ_r_ order, as in Figure 3b, the nonbonded potential *V*_nb_ contains negative regions. For soft models, where particles overlap, potentials with negative parts can create instabilities:^40,41^ molecules in the system might agglomerate and collapse into a small region of space. Here, due to the complex functional form of *V*_nb_, the formal stability of the system cannot be ensured for individual monomers. In practice, however, it is the entire potential surface originating from groups of bonded monomers that is important, rather than pair interactions, and the combination of the fixed bond length with the bonded potentials tends to discourage collapse. We also always choose κ, λ, and ζ such that *V*_lamella_ remains positive.^7^ Therefore, the negative regions of *V*_nb_ are only located in the ring caused by *V*_reg_, and as these are away from the interaction centers, the likelihood of collapse is probably reduced. We see no evidence of collapse in any of our simulations, which are carried out at a range of different densities and include building up the structure from a disordered state. Prior research^42−45^ into liquid crystals has also been carried out using soft core Gay–Berne potentials with negative regions away from the center, without reporting collapse.

### 2.3 System Setup and Monte Carlo Sampling

The systems are initially set up in monodomains in boxes with edge lengths *L*_α_ (where α = *x*, *y*, *z*) and periodic boundary conditions (PBC) in each direction. Strictly monodisperse molecules in the all-trans conformation are evenly distributed among *n*_*y*_ lamellae; the normals of the lamellae are parallel to the *y*-axis. The chains in each lamella are placed into *n*_*z*_ equally spaced stacking layers. Each of them contains the same number of molecules, *n*_*x*_. Chains lie with their backbones, that is, the ***n***_*j*_^(1)^(*s*) of the monomers, parallel to the *x*-axis and stacked along the *z*-axis (i.e., ***n***_*j*_^(3)^(*s*) is parallel to the *z*-axis). Importantly, there is no correlation across different stacking layers regarding the position of chains along the *x*-direction. Note that *n*_*x*_, *n*_*y*_, and *n*_*z*_ refer to the organization of molecules in the initial setups; the arrangements can change during the simulations. For each system in the rest of the paper, we quote *n*_*x*_, *n*_*y*_, and *n*_*z*_ for the initial configurations.

Subsequently, Metropolis Monte Carlo (MC) simulations are used to equilibrate the systems and sample the configuration space. We use two ensembles: standard canonical and isostress. In both ensembles, the volume *V* and, therefore, the average density ρ_0_ = *nN*/*V* are fixed. We mostly use ρ_0_ = 2.05 monomers/σ^3^, which in isotropic melts corresponds to a packing fraction of monomers of Φ ≈ 8.58, where Φ = ρ_0_*v*_0_. These choices of ρ_0_ and Φ correspond to rather moderate values among the coarse-grained densities and packing fractions one finds when representing real polymers using our drastically coarse-grained approach. For example, using one single coarse-grained site to represent one repeat unit, we estimate that for poly(3-hexylthiophene), poly(3-dodecylthiophene), PE12 polyester,^3^ and Ac-Ndc_9_-Nte_9_ polypeptoid,^8^ ρ_0_ ≈ 1.6, 8.6, 3.4, and 6.5, respectively. The corresponding packing fractions are Φ ≈ 6.8, 36, 14, and 28. In Section 1 of the Supporting Information, we explain how these estimations are made. Other choices of ρ_0_ are considered in section 3.2 when exploring the stability of the Σ_r_ mesophase. The temperature *T* enters implicitly via the coefficients governing the strength of the potentials (they are defined in units of *k*_B_*T*, see section 2.2).

Two MC moves are employed for simulations in the canonical ensemble: reptation^46,47^ and “flip”^48^ (also known as “crankshaft”^49^) with 80% and 20% probability, respectively. The reptation move is modified to account for the presence of ghost bonds, as described previously.^19^

Flip moves are performed differently depending on whether a real or ghost monomer is randomly selected. If a real monomer is chosen, it is rotated by a random angle (from a uniform distribution between −180° and 180°) around a local axis joining the positions of the previous and next monomers of the chain (even if one of these is a ghost). If a ghost monomer is chosen, its connecting ghost bond is rotated by a random angle around the axis defined by the bond immediately next to it.

Simulations in the isostress ensemble also include a third, variable-shape-constant-volume^50−53^ (VSCV) MC move with 0.1% probability. This move optimizes the box dimensions *L*_α_ to make them commensurate with the natural geometry of the lamellar structure, under the constraint of constant volume *V* (and, therefore, constant average density ρ_0_). We emphasize, however, that the move maintains an orthogonal shape of the box and does not optimize the angles between its edges (in contrast, e.g., to the Andersen–Parrinello–Rahman barostat^54^ in Molecular Dynamics). The application of the VSCV algorithm has been described in detail previously^7^ for a similar system. Proposed new values for *L*_α_ are chosen randomly, and the first real monomer (i.e., not a ghost) of each chain undergoes an affine transformation so that its coordinates maintain their original proportions relative to the box lengths *L*_α_. The other monomers are translated to preserve internal structures (bond lengths, bond angles, dihedral angles) and orientations that the chains had immediately before the move. Here, the maximum allowed changes to *L*_*y*_ and *L*_*z*_ are ±0.05; *L*_*x*_ is then determined by the constant volume constraint.

Because the VSCV move is computationally costly, we use the isostress ensemble only for small systems where *n* ≤ 400. Simulations of larger systems are performed in the standard canonical ensemble. In this case, *L*_α_ are set to optimum values commensurate with periodicities (lamellar and stacking spacings) estimated from isostress simulations of smaller systems ({*n*_*x*_, *n*_*y*_, *n*_*z*_} = {2, 8, 25}). For each of these smaller systems, we perform eight simulations starting from configurations with different initial box dimensions *L*_α_. The runs continue until the dimensions of all eight simulations converge to similar values of *L*_α_.

## 3 Results and Discussion

### 3.1 Structure of the Σ_r_ Mesophase

When the strengths of all terms in *V*_nb_ are sufficiently high, our model forms a Σ_r_ mesophase for all chain lengths studied (see section 3.2). Here we explain the structure of this mesophase for one representative chain length *N* = 16 and one choice of parameters κ = 0.874, λ = 0.408, ζ = 0.204, η = 1, θ_0_ = 150°, and ρ_0_ = 2.05.

To facilitate structural analysis, we equilibrate Σ_r_ monodomains where the chain backbones, lamellar spacing, and stacking direction are oriented along the *x*-, *y*-, and *z*-axis of the simulation box, respectively. An approximately cubic box with dimensions {*L*_*x*_, *L*_*y*_, *L*_*z*_} = {25.10, 21.36, 20.98} and *n* = 1440 chains ({*n*_*x*_, *n*_*y*_, *n*_*z*_} = {3, 12, 40}) is used to investigate the general structure of the mesophase. Highly asymmetrical boxes, which are long in one dimension α = *x*, *y*, or *z*, and short in the other two, are used to investigate correlation functions along each direction. Specifically, we use {*L*_*x*_, *L*_*y*_, *L*_*z*_} = {133.89, 7.12, 7.87}, {16.74, 56.96, 7.87}, and {16.74, 7.12, 62.94}. Each box contains *n* = 960 chains, with {*n*_*x*_, *n*_*y*_, *n*_*z*_} = {16, 4, 15}, {2, 32, 15}, and {2, 4, 120}, respectively. We study configurations prepared with 16 independent simulations (repeats).

**Figure 4:**
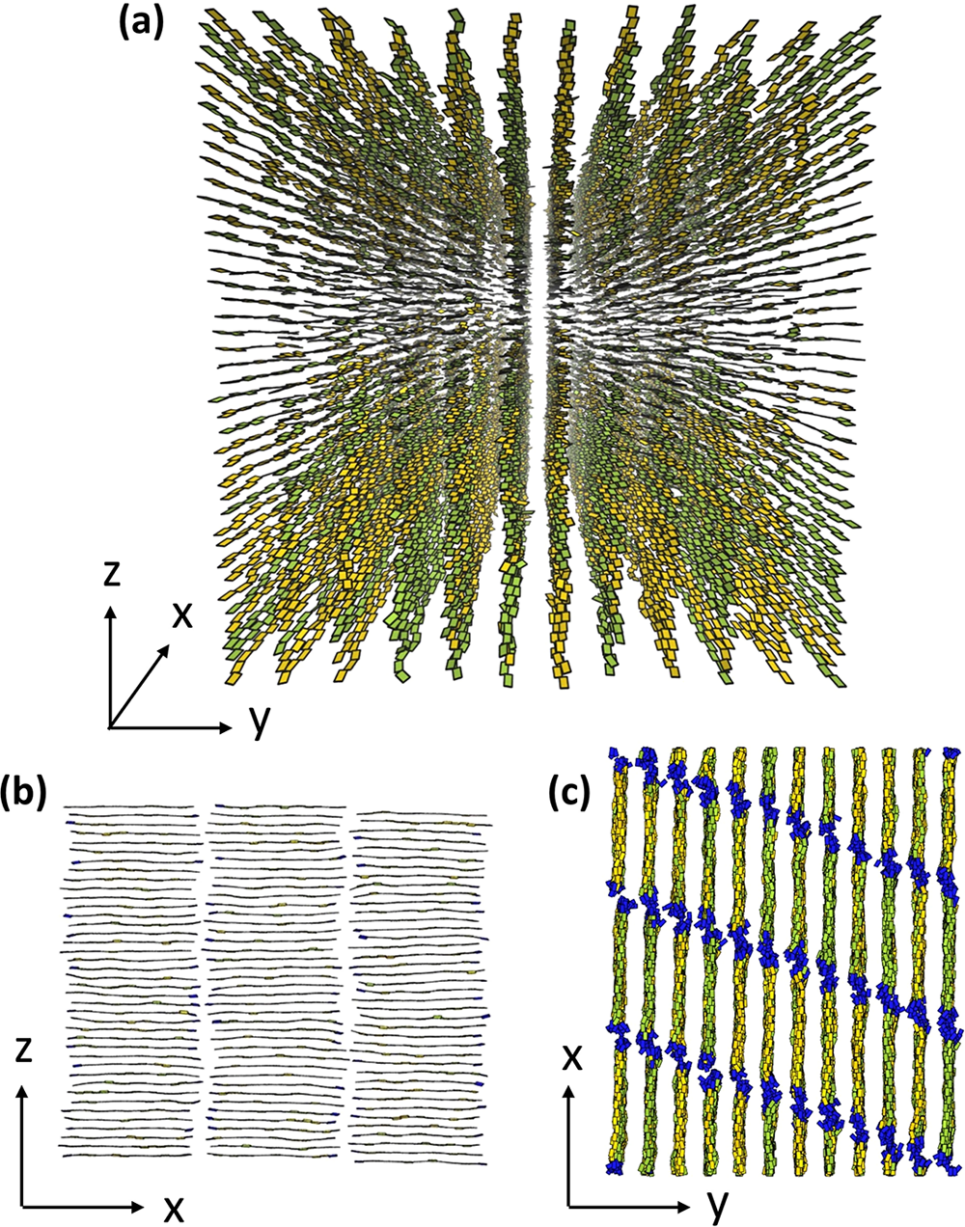
Visualizations of the Σ_r_ mesophase produced by simulation, where each board shows a monomer in its current orientation. (a) Perspective view of an entire simulation box showing a collection of regular lamellae. (b) Side view of a single lamella, demonstrating the intralamellar SmA structure. (c) Top-down view of a collection of lamellae, where the end monomers are colored in blue to demonstrate the interlamellar SmC structure.

A lamellar arrangement is evident in Figure 4a, which presents a typical 3D configuration from our MC simulations. Figure 4b and c are side views of this configuration along the *y*- and *z*-axis, respectively. In such 3D configurations, we quantify order along each direction α by calculating 1D pair-correlation functions *g*(*r*_α_) defined as
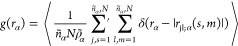
10The prime on the second sum excludes monomer self-interactions, that is, *m* ≠ *s* for *l* = *j*. Furthermore,·*r*_*jl*;α_(*s*, *m*) are projections of distance vectors between monomers onto the direction α, and *ñ*_α_ is the number of chains contributing to the calculation of *g*(*r*_α_). ρ̃_α_ = (*ñ*_α_ – 1)*N*/2*L*_α_ is a normalization factor. *g*(*r*_*x*_) probes correlations along the chain backbone direction, where *ñ*_*x*_ stands for the number of chains located in the same lamella. For *g*(*r*_*y*_), which probes order in the direction orthogonal to the lamellae, *ñ*_*y*_ is the total number of chains in the system, that is, *ñ*_*y*_ = *n*. Finally, *g*(*r*_*z*_) quantifies correlations along the stacking direction *z*; in this case, *ñ*_*z*_ is the number of chains in the same stack. For *g*(*r*_*x*_) and *g*(*r*_*z*_), the angular brackets in eq 10 indicate canonical averaging over all lamellae and all stacks, respectively. For *g*(*r*_*y*_), the angular brackets represent canonical averaging over all equilibrated configurations with the same size and shape. We obtain 1D pair-correlation functions as histograms with a bin size of 0.5 for *g*(*r*_*x*_) and 0.01 for *g*(*r*_*y*_) and *g*(*r*_*z*_).

**Figure 5:**
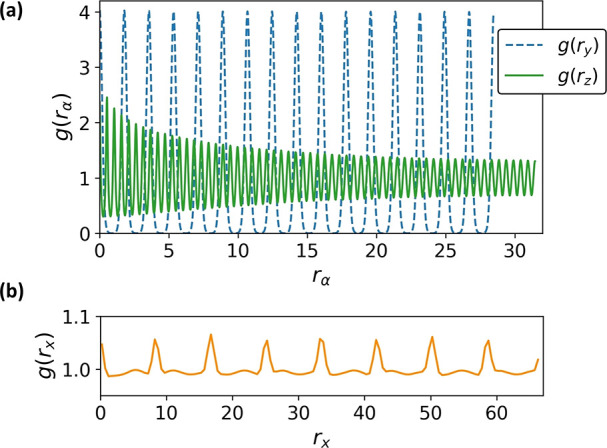
(a) 1D pair correlation functions *g*(*r*_*y*_) (dashed line) and *g*(*r*_*z*_) (solid line) calculated along the interlamellar and stacking directions, respectively. (b) 1D pair correlation function *g*(*r*_*x*_) calculated along the backbone direction.

The *g*(*r*_*y*_) presented in Figure 5a (dashed line) demonstrates that the positional order orthogonal to the lamellae is indeed strong: peaks are regularly spaced and there is no perceptible decay in their height. The spacing of the peaks corresponds to a lamella period of *d*_lam_ = 1.78; this value changes as the model parameters are varied.

Translational invariance is also broken within each lamella, as demonstrated by Figure 4b, which provides a side view of a representative lamella from the configuration in Figure 4a. Within the lamellar *xz*-plane, chains form stacks analogous to a “quasi-2D” Smectic A (SmA). The periodic shape of *g*(*r*_*x*_) in Figure 5b (calculated in the system with *L*_*x*_ accommodating 16 chain backbones) is consistent with SmA order and suggests that correlations along the backbone direction do not decay with distance within each lamella, at least for the considered system sizes.

Apart from lamellae and chain registration, Figure 4b demonstrates that our model reproduces another hallmark of Σ_r_ mesophases: periodicity along the stacking direction. This periodicity is quantified by the *g*(*r*_*z*_) shown in Figure 5a (solid line) for the system accommodating 120 stacking layers. The peaks are regularly spaced, demonstrating a regular stacking distance *d*_st_ = 0.525, but their heights decay with distance, suggesting that only quasi long-range order^55^ (QLRO) is present along the stacking direction. The rate of decay also increases with *L*_*z*_, when comparing simulation boxes of different shapes, as expected^56^ for QLRO (see Section 2 of the Supporting Information).

Interestingly, the top view in Figure 4c demonstrates that the positions of polymer chains across different lamellae in the 3D configuration are correlated. The ends of the chains are shown in a different color than the internal monomers, indicating that the mesophase forms an analogue of Smectic C (SmC) in the *xy*-plane. The tilt direction is generally consistent across the simulation box, but tilt reversals sometimes occur, producing zigzags. These stem from metastability: multiple domains frequently form during the early stages of equilibration, particularly when the initially developed tilt angle cannot be maintained across the whole simulation box due to the PBC. It is observed that the tilt angles adjust during the simulations to reduce discontinuities.

Although the Σ_r_ mesophase of our model is well structured on the level of polymer chains, it has substantial disorder on the monomer scale in some directions. We quantify monomer packing using the 3D correlation function:
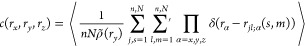
11where
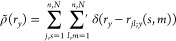
12We calculate *c*(*r*_*x*_, *r*_*y*_, *r*_*z*_) by binning the distances between all pairs of monomers in a configuration into a histogram, with bin size 0.05 in the *x* and *z* directions and 0.1 in the *y* direction. Averaging takes place over all configurations with the same tilt angle.

**Figure 6:**
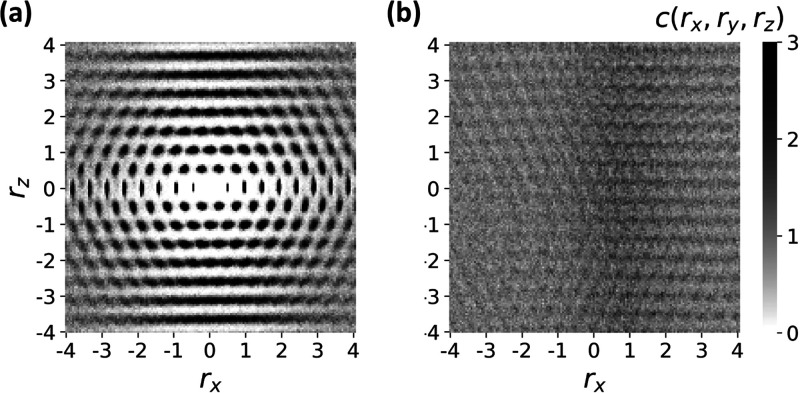
Contour plots of the positional correlation function *c*(*r*_*x*_, *r*_*y*_, *r*_*z*_) showing (a) intralamellar monomer correlations, *c*(*r*_*x*_, 0, *r*_*z*_), and (b) correlations between monomers in neighboring lamellae, *c*(*r*_*x*_, *d*_lam_, *r*_*z*_). Both graphs are plotted in the lamellar *xz*-plane. The scale for *c*(*r*_*x*_, *r*_*y*_, *r*_*z*_) has a cutoff of 3, so spikes with values higher than 3 are also shown in black.

Figure 6a presents a contour plot of the 2D slice of *c*(*r*_*x*_, *r*_*y*_, *r*_*z*_) that corresponds to intralamellar correlations, that is, *r*_*y*_ = 0. It demonstrates that the monomers locally form an analogue of a 2D centered rectangular lattice. Further away, their correlations are washed out by thermal fluctuations. In Figure 6b, we plot the 2D slice of *c*(*r*_*x*_, *r*_*y*_, *r*_*z*_) corresponding to monomers found in neighboring lamellae, that is, *r*_*y*_ = *d*_lam_. Here, only a faint lattice can be discerned, suggesting weak *xz*-positional correlations between monomers in different lamellae. A broad vertical dark stripe, indicating higher probability, appears slightly to the right of the origin. It corresponds to the SmC shift of chain backbones along the *x* direction between subsequent lamellae. When we average over configurations with different tilt angles, we observe that interlamellar correlations are washed out, confirming that they are indeed very weak.

The intralamellar monomer-level lattice has a strong energetic driver based on minimizing *V*_reg_, see Section 3 of the Supporting Information for details. The intralamellar SmA order forms very quickly in our simulations, and there is evidence that there is a strong energetic contribution to its appearance, although there may also be entropic effects. The origin of the SmC order is less clear, because we were not able to find clear evidence of an energetic benefit. Further discussion can be found in Section 4 of the Supporting Information.

Knowing the structure of our Σ_*r*_ mesophase enables a back-of-the-envelope calculation. The volume per chain in the Σ_*r*_ mesophase is *d*_st_*d*_lam_[(*N* – 1)*b̃* + Δ]. Here, *b̃* = *b* sin(θ/2) and Δ is the intralamellar “gap” between SmA stacks. Hence, (*N* – 1)*b̃* + Δ approximates the characteristic length of one SmA stack. Of course, this simple estimate neglects conformational fluctuations (deviations from the all-trans state), as well as possible chain backfolding, and cross-lamellar bridging. Considering that the volume per chain also equals *N*/ρ_0_, we obtain
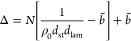
13In a realistic model, Δ should remain “microscopic”, even for long chains, that is, Δ must saturate with *N*. This requirement holds when:
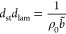
14Equations 13 and 14 lead to two conclusions. First, they suggest that the range of densities where our Σ_*r*_ mesophase maintains realistically small gaps is rather narrow. We refer to a range of densities because *d*_st_ and *d*_lam_ are not constant, but functions of ρ_0_ (and *N* for short chains). Second, they guide the choice of *R*_2_. In a stable sanidic mesophase, lamellae must interact. Therefore, the range of our potential provides an upper boundary for *d*_lam_, that is, *d*_lam_ ≤ 2. Assuming that *d*_st_ ≤ *R*_2_, we obtain from eq 14 that *R*_2_ ≥ 1/2ρ_0_*b̃*. Thus, our basic choices of density ρ_0_ = 2.05 and θ = 150° correspond to *R*_2_ ≥ 0.5. Starting from this value, we increased *R*_2_ in our exploratory scans and found that *R*_2_ = 0.65 led to clear-cut Σ_*r*_ structures.

### 3.2 Robustness of Σ_r_ Mesophase

Here, we consider the robustness of the Σ_r_ mesophase against varying the strengths of the nonbonded potentials, chain length *N*, preferred bond angle θ_0_, and average density ρ_0_.

To investigate the effect of the nonbonded potentials, we construct a phase diagram. We fix the strength of the isotropic repulsion *V*_iso_ to κ = 0.874 and vary the other coefficients λ, ζ, and η. The coefficients λ and ζ are allowed to change within a range that maintains a positive potential for lamellar order *V*_lamella_ > 0. This condition ensures that β*V*_nb_ is always repulsive when monomers come close, that is, *r*_*jl*_(*s*, *m*) ≃ 0 (see section 2.2). All other parameters are fixed to *N* = 16, θ_0_ = 150°, and ρ_0_ = 2.05. We perform simulations of systems with *n* = 400 chains ({*n*_*x*_, *n*_*y*_, *n*_*z*_} = {2, 8, 25}) in the isostress ensemble. To construct the phase diagram, we combine visual inspection with several quantitative indicators. The transition from an isotropic melt to biaxial nematic *N*_b_ is identified using uniaxial and biaxial order parameters, *S* and *B*, calculated in a standard way^57^ (see Section 5.1 of the Supporting Information). We consider that *N*_b_ is formed when both *S* and *B* are ≥0.5. The transition into a Σ_d_ disordered lamellar mesophase is characterized by the occurrence of regular peaks corresponding to lamellar spacing in *g*(*r*_*y*_), the 1D correlation function introduced in eq 10.

A main characteristic of the transition from Σ_d_ to our highly ordered Σ_r_ mesophase is the appearance of SmA order within each lamella, which we quantify using a standard-order parameter^35,58,59^ Λ (see Section 5.2 of the Supporting Information). For the system sizes considered here, we find that Λ > 0.7 is a strong indicator of SmA order; visual corroboration is required when Λ ≈ 0.7. The presence of cross-lamellar SmC is mainly judged by eye, although a method for quantification is discussed in Section 5.3 of the Supporting Information.

**Figure 7:**
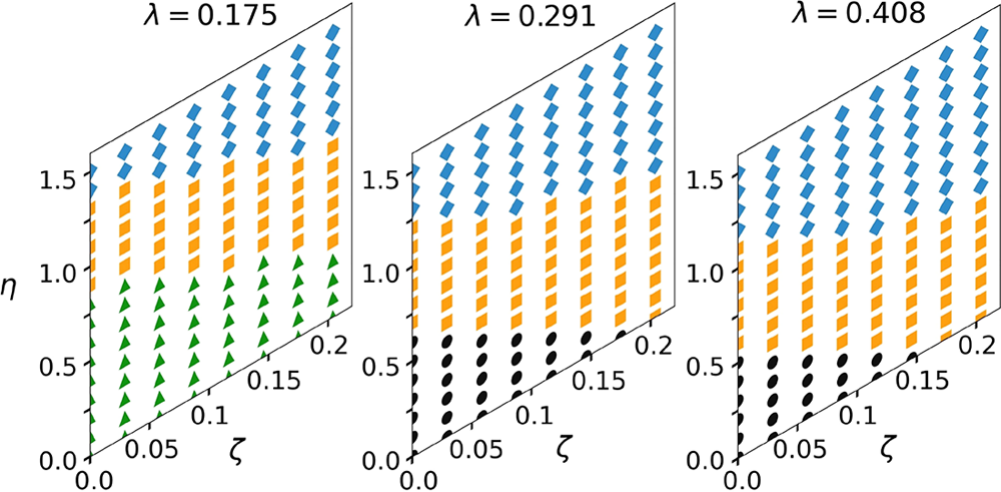
Dependence of phase behavior on the strength of different components of the nonbonded potential β*V*_nb_. Data points correspond to isotropic melt (green triangles), biaxial nematic *N*_b_ (black circles), Σ_d_ sanidic (orange squares), and Σ_r_ sanidic (blue diamonds). *N* = 16 and κ = 0.874 throughout.

Figure 7 presents the phase diagram, where data points correspond to an isotropic melt, a biaxial nematic *N*_b_,^19^ a Σ_d_ sanidic mesophase,^7^ and our Σ_r_ sanidic mesophase. We emphasize that this diagram is qualitative because of the limited amount of repeats and no systematic study into finite size effects. Moreover, we use a compressible model within a statistical ensemble where two extensive variables, total number of particles and system volume, are fixed. Therefore, the identification of phase boundaries is complicated by the possibility of phase coexistence.^60^ Nevertheless, Figure 7 is sufficient to demonstrate the stability of the Σ_r_ mesophase over a broad range of parameters. Overall, Σ_r_ is encouraged when λ, ζ, and η are high. Interestingly, however, a Σ_r_ mesophase also appears when ζ = 0, provided that λ and η are sufficiently large. Although no upper phase boundary for Σ_r_ is identified, we note that high values of η can prohibitively slow down equilibration.

Although each of the four components of *V*_nb_ is mainly responsible for an individual feature of structural order, we emphasize that there is a cooperativity between them (which is also influenced by chain connectivity). Therefore, it is not straightforward to develop simple guidelines for choosing values λ, ζ, or η that promote specific mesophases, by considering only the isolated effects of the interactions that are conjugated to each of these parameters. Nevertheless, we provide some qualitative analytical estimations in Section 6 of the Supporting Information.

Changing other system parameters also preserves the characteristic structural features of our mesophase. In particular, we do not observe any qualitative changes when the most probable bond angle is varied within the range θ_0_ = 140–170°, despite expectations that straighter chains might produce an interlamellar SmA rather than SmC. For example, in another study,^35^ hard zigzag molecules were unable to form SmC for θ_0_ > 154° (we clarify, however, that in that case there were no lamellae and the order was driven purely by steric interactions).

In general, chain length parity can affect smectic behavior.^61−63^ For board-like molecules, an odd–even effect has been recently reported in simulations of nonlamellar oligo-thiophenes, where only even-numbered chains formed SmC mesophases.^64^ Therefore, we perform simulations for consecutive chain lengths, *N* = 13, 14, 15, and 16, with all other parameters fixed (κ = 0.874, λ = 0.408, ζ = 0.204, η = 1, θ_0_ = 150°, and ρ_0_ = 2.05). For our model, we observe no odd–even effect: all aforementioned chain lengths produce mesophases with the same symmetries as described in section 3.1.

We find that the structure of our Σ_r_ mesophase is qualitatively insensitive even to pronounced variations in chain length; namely, when we compare mesophases composed of molecules with *N* = 16, 24, and 32. Of course, the largest of these chains, *N* = 32, is only about five times longer than the persistence length of the ideal chain. It is plausible that much longer chains will destabilize (at least) some of the features of our Σ_r_ order, for example, because of increased conformational “defects”. Exploring this issue requires additional MC moves that enable equilibration of long polymers, such as rebridging^65,66^ and configurational bias^67^ algorithms.

Finally, we check the qualitative prediction, eqs 13 and 14, that our Σ_r_ mesophase exists in a limited range of densities. For three different chain lengths, *N* = 16, 24, and 32, we consider the same point in the parameter space (κ = 0.874, λ = 0.408, ζ = 0.204, η = 1, and θ_0_ = 150°) and vary the average system density ρ_0_. For this purpose, simulations are performed in the isostress ensemble, with a constant number of chains *n* = 270, but different volumes ({*n*_*x*_, *n*_*y*_, *n*_*z*_} = {3, 6, 15}).

**Figure 8:**
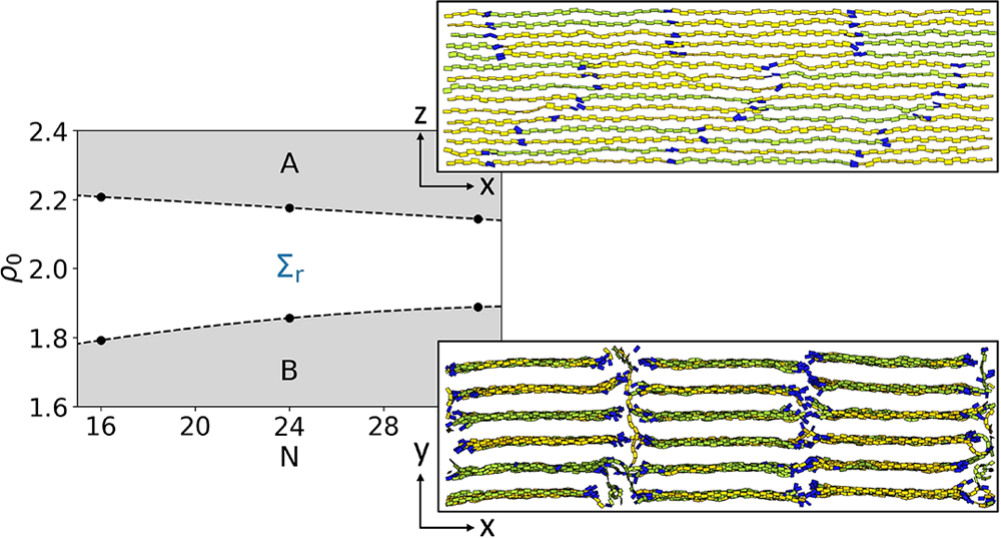
Qualitative stability diagram for the Σ_r_ mesophase as a function of density and chain length. The boundaries represent the first clear example of Σ_r_ (according to the structure in section 3.1) when approaching from regions “A” and “B”. Insets are visualizations of representative molecular arrangements from each of these regions: the visualization for “A” shows a single lamella from the side, whereas “B” shows a top-down view of a collection of lamellae.

Figure 8 presents a qualitative stability diagram of the Σ_r_ mesophase as a function of ρ_0_ and *N*. Indeed, for each *N*, the Σ_r_ mesophase is stable in a rather limited range of densities, marked as the “Σ_r_” region. Outside this range, in regions “A” and “B”, some of the Σ_r_ features start to disappear.

In region “A”, gaps between stacks are eliminated and chain ends overlap along the backbone direction (see upper inset in Figure 8), meaning that Δ < 0. Presumably, this overlap at high densities stems from the softness of interactions and is favored by the negative part of *V*_reg_. In principle, the Σ_r_ mesophase may actually extend to higher densities: the VSCV move optimizes only the lengths *L*_α_ while maintaining the orthogonal shape of the simulation box. If the box angles were also allowed to adjust, the system might be able to maintain Δ > 0 until somewhat higher densities by tilting the lamellae. Δ < 0 occurs at slightly lower densities as *N* increases, in agreement with eq 13 (see decreasing boundary of region “A”).

In region “B”, gaps are large due to low density. As Δ increases, more disorder around chain ends and bridging between lamellae occur. We set the boundary of the “B” region to densities where chain ends start to bridge; an example is shown in the lower inset of Figure 8. Of course, for some ρ_0_, we might enter a regime of average system densities for which Σ_r_ coexists with a less-ordered phase. In agreement with eq 13, we observe that large Δ occur at slightly higher densities as *N* increases (see increasing boundary of region “B”).

In summary, we find that our Σ_r_ mesophase is robust against varying parameters such as nonbonded potential strengths, chain length, and bond angle. As expected from section 3.1, the range of possible densities of the Σ_r_ mesophase formed in our soft model is rather limited.

### 3.3 Scattering and Relevance to Actual Sanidics

We now compare the structure of our Σ_r_ mesophase with two highly ordered sanidics that have been reported in experiments.^3,8^ In these studies, the molecular organizations of the mesophases were determined from scattering measurements. In our particle-based simulations, molecular organizations are known without the need to perform scattering calculations, because monomer coordinates are explicitly available. However, calculating scattering patterns and juxtaposing them with experimental data offers an illustrative framework for performing structural comparisons. Because of the drastic coarse-graining used in our model, we know a priori that our scattering patterns cannot quantitatively reproduce properties sensitive to atomistic details, such as atomic form factors. Furthermore, in this work, our model is not tailored to represent a specific material. Therefore, we cannot directly compare absolute length scales associated with scattering peaks in our simulations and experiments. For the same reason, the ratios of lengths characterizing different geometric features of mesophases in our generic model, such as stacking distance, lamellar spacing, and smectic period, can differ quantitatively from the ratios found in a real material. Hence, the scattering peaks in our patterns can appear at somewhat different positions relative to each other than in an actual scattering pattern. The goal of our discussion is to illustrate structural similarities and differences between simulations and experiments through the presence (or absence) of scattering signals that are linked to certain generic features of molecular organization in the mesophases.

We calculate scattering from sanidic monodomains, equilibrated and oriented as described in sections 2.3 and 3.1. In general, experimental samples are expected to have multiple domains with some disorder in between, caused, for example, by slow kinetics of ordering. Hence, scattering patterns calculated from monodomains may indicate stronger long-range order (and therefore more higher order scattering peaks) than experimental data. Monodomains are, however, well-defined equilibrium systems. In contrast, the preparation of nonequilibrium samples with experimentally relevant polydomain structures in simulations would require a realistic description of the kinetics of ordering. Accounting for the kinetics of structure formation is beyond the scope of the current study, which is focused on initial model development.

**Figure 9:**
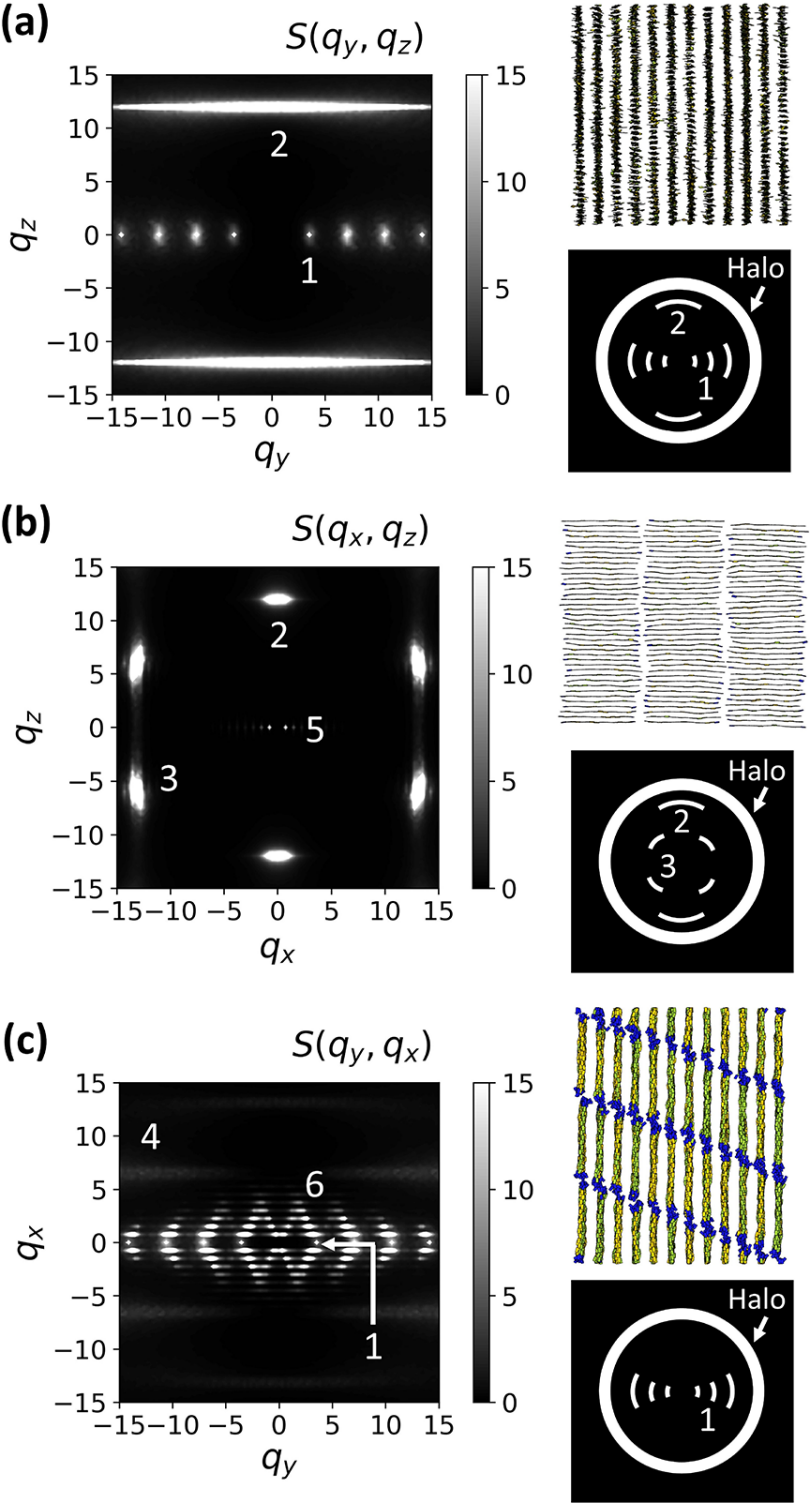
2D scattering patterns of our Σ_r_ mesophase obtained from simulations with *N* = 16, in the (a) interlamellar-stacking *yz*, (b) backbone-stacking *xz*, and (c) interlamellar-backbone *yx* planes. There is a cutoff for the scales of the plots, so peaks higher than 15 are shown in white. Insets for each image show visualizations of the relevant scattering planes (replotted from Figure 4), and cartoons based on sketches introduced by Ebert et al.^3^ as a distilled representation of the key features of their scattering data for PE12 (note that we transform the original sketches of Ebert et al.^3^ into the same coordinate frame as the simulation data).

We consider monodomains accommodated in approximately cubic simulation boxes, {*L*_*x*_, *L*_*y*_, *L*_*z*_} = {25.10, 21.36, 20.98}, each containing *n* = 1440 chains of length *N* = 16 ({*n*_*x*_, *n*_*y*_, *n*_*z*_} = {3, 12, 40}). 2D scattering functions are calculated using
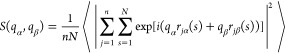
15Here, α and β refer to mutually exclusive combinations of the *x*, *y*, and *z* components of the scattering vector ***q***. The components comply with the PBC, that is, *q*_α_ = 2π*m*/*L*_α_ (α = *x*, *y*, *z*), where *m* is an integer. Angular brackets indicate averaging over different configurations and repeats. Figure 9 shows the calculated 2D scattering patterns; to facilitate their interpretation, we also replot visualizations of the planes they represent from Figure 4. We report scattering vectors in units of σ^–1^.

**Figure 10:**
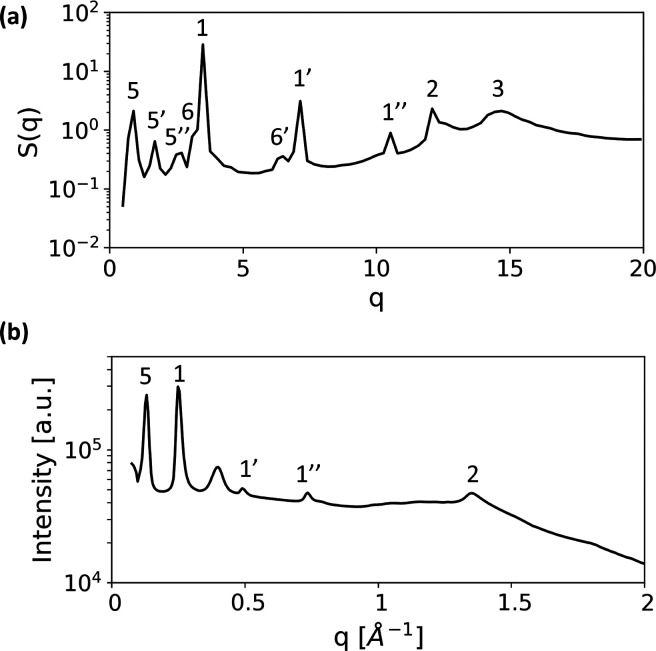
(a) 1D spherically averaged scattering pattern of our Σ_r_ mesophase obtained from simulations with *N* = 16. (b) Powder diffraction pattern experimentally measured for the polypeptoid Ac-Ndc_9_-Nte_9_ at 50 °C. The data used to prepare the plot have been published by Greer et al.^8^ (see Figure S5 of the Supporting Information of ref (8)). The unlabeled peak arises from the sample holder.

To obtain powder diffraction spectra *S*(*q*), where *q* = |***q***|, we first calculate the complete 3D scattering function:
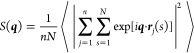
16The components of the scattering vector ***q*** comply with the PBC, as described before. *S*(*q*) is obtained by spherically averaging the data for *S*(***q***) and is presented in Figure 10a. The triangular-like shapes of some peaks result from the quantized values of ***q*** for which *S*(***q***) is available.

To compare our scattering patterns with experiments, we focus on two studies.^3,8^ The first study by Ebert et al.^3^ reported a Σ_r_ mesophase in a rigid-rod polyester comprising aromatic backbones and alkyl side-chains, that is, the PE12 sample.^3^ To obtain 2D scattering patterns, they used flow to prepare macroscopic monodomains oriented along well-defined directions and, in this sense, their samples are analogous to the idealized set up of monodomains used in our simulations. Ebert et al.^3^ distilled the key features of their scattering data into sketches, which they then used to discuss the basic characteristics of molecular arrangements in their sanidic mesophases. The generic nature of these sketches makes them particularly suitable for comparison with the scattering patterns from our generic model. In Figure 9, we replot the sketches provided by Ebert et al. for the PE12 sample (Figure 3 of ref (3)), so that the orientations of the mesophase directors in our simulations and their experiments are consistent.

The second study by Greer et al.^8^ reported a sanidic mesophase involving polypeptoid diblock copolymers. 1D X-ray scattering patterns were obtained for their multidomain samples, and the authors inferred the structure of the mesophase from careful analysis of this data. Greer et al.^8^ did not explicitly mention that their sanidic mesophase is Σ_r_, but we believe the structural features they found indicate that this is the case. In Figure 10b, we replot the data for this mesophase (Ac-Ndc_9_-Nte_9_ at 50 °C); the original data are available in Figure S5 of the Supporting Information of ref (8).

One important qualitative difference between the sketches for PE12 and simulations is the lack of amorphous halo in the latter. This is not surprising; the halo in experimental data stems from scattering from amorphous side chains, which our model does not include explicitly, although backmapping^68^ could be used to add them a posteriori.

Intense narrow reflections, labeled “1”, appear in Figures 9a,c and 10 in simulations and both experiments. They are attributed^3,8^ to a highly regular lamellar spacing. In all cases (sketches of Ebert et al.,^3^ 1D scattering patterns of Greer et al.^8^ and simulations), first and higher order peaks are evident. To avoid crowded 2D scattering patterns, we label only one representative primary peak in each group of reflections; in 1D patterns, secondary and tertiary reflections are indicated by the same label as the primary peak but with primes, for example, “1′” and “1′′”. Compared to the primary peak “1”, the intensities of the secondary and tertiary reflections in the 1D patterns of Greer et al.^8^ are weaker than in our case; this difference might stem from the multidomain structure of the experimental samples.

The peaks “2” in the scattering patterns of our mesophase (Figure 9a,b) stem from regular stacking. Similar peaks explained^3,8^ by regular stacking were also identified in the scattering patterns of Ebert et al.^3^ and Greer et al.^8^ (Figures 9a,b and 10). Comparing with the sketches of Ebert et al.,^3^ we observe one qualitative difference. For our mesophase, the “2” peaks are sharp along *q*_*z*_ due to a strong stacking periodicity, but very elongated along *q*_*y*_ (Figure 9a). This feature indicates that, in simulations, stacks in neighboring lamellae can shift arbitrarily with respect to each other along *z*; this is consistent with the weak interlamellar correlations reported in Figure 6b. Ebert et al.^3^ did not report elongation of “2” peaks along *q*_*y*_, but from their general discussion, it remains unclear whether stacking is correlated between lamellae in PE12. For the polypeptoids, the stacking is uncorrelated, similar to our mesophase. In that system, the absence of cross-correlation is suggested by the loss of interlamellar-stacking cross-peaks observed^8^ when melting from crystal.

The scattering from our mesophase has cross-peaks “3” in the *q*_*x*_–*q*_*z*_ plane (Figure 9b) which arise from the intralamellar monomer-level lattice (registration) observed in Figure 6a. Analogous cross-peaks corresponding to intralamellar registration of monomers were identified^3^ in the PE12 scattering pattern; see sketch in Figure 9b. The experiments on polypeptoids did not provide any evidence that those polymers are mutually registered on a monomer level along the backbone direction (note that this lack of registration does not contradict the presence of periodicity along the stacking direction described previously).

At first sight, the faint broad cross-peaks “4” in our data (visible in Figure 9c, and responsible for raising the baseline at *q* > 8 in Figure 10a) appear to imply monomer-level registration between molecules in different lamellae. However, these peaks are actually part of the single-chain structure factor, so do not indicate cross-lamellar correlations. Similar to our mesophase, no interlamellar monomer-level registration has been identified (so far at least) in the PE12 or polypeptoid systems.

In our mesophase, chains are organized within each lamella into SmA layers (see section 3.1), which are signaled by the scattering peaks “5” and their secondary reflections (Figures 9b and 10a). The polypeptoid system shows a prominent reflection (also marked as “5” in Figure 10b), which was assigned^8^ to the length of chain backbone. We hypothesize that this signal is too strong to be attributed solely to the single-chain structure factor and, therefore, signifies smectic order analogous to that found in our mesophase. This smectic order might be driven by microphase separation between the blocks of the diblock copolymer. In other words, although polypeptoid backbones can shift with respect to each other on the level of monomers, they remain registered when observed on the scale of entire chains. Smectic peaks are not evident, and were not discussed,^3^ in scattering patterns of PE12. Still, we believe that their absence does not completely exclude smectic order in PE12 because: (i) These scattering features could be present at *q*-values that were not accessed in the experiments. (ii) The samples were prepared under nonequilibrium conditions and flow might have inhibited smectic order.

The cross-peaks “6” in Figures 9c and 10a are the only signals in the scattering patterns from our simulations that do not have an equivalent scattering feature reported^3,8^ in at least one of the two experimental systems. They arise from chain-level SmC registration between molecules in different lamellae (see section 3.1). For a SmC with a single tilt direction, the peaks would lie on a single diagonal extending from each lamellar spacing reflection “1”. However, averaging over systems with opposite tilting produces the characteristic diamond shapes. These interlamellar smectic peaks occur only weakly in the 1D plot (Figure 10a), as a shoulder to the primary lamellar spacing peak “1”. This “merging” of scattering features suggests that intermolecular smectic order cannot be completely ruled out for the polypeptoid system, based on powder diffraction spectra only.

In summary, these structural comparisons suggest that there are at least two different flavors to the Σ_r_ mesophases observed in experimental systems. Namely, backbone registration within lamellae can occur either on the level of monomers, for example, in PE12,^3^ or chains, for example, in polypeptoid diblock copolymers.^8^ In experiments, no correlations were observed between monomers or chains located in different lamellae. Our model exhibits both monomer and chain-level registration within lamellae, as well as chain-level registration between lamellae. Therefore, it appears to be more highly ordered and closer to crystalline in structure than any of the yet observed experimental sanidic mesophases.

## 4 Conclusions

We developed a generic coarse-grained model enabling Monte Carlo simulations of Σ_r_ sanidic mesophases of board-like polymers. The hallmarks of these highly ordered mesophases are as follows:^1,3^ they have parallel lamellae, which are created by the assembly of polymer backbones into stacks. These lamellae are separated by layers of disordered side chains. In each lamella, polymers stack at regular distances, forming a periodic structure along the stack normal. Furthermore, there is positional order along the long axis of each stack, that is, polymers register along the direction of chain backbone.

In our approach, polymers are represented by a hindered-rotation-chain model, where variations of angular and torsional degrees of freedom are subjected to a generic energy landscape. Nonbonded interactions between coarse-grained monomers are described by anisotropic potentials that are “soft”, namely, their strength is comparable to the thermal energy. To define them, we built upon generic anisotropic “force fields” developed previously for modeling biaxial nematic^19^ and disordered lamellar sanidic^7^ Σ_d_ mesophases in conjugated polymers. Here, to model a Σ_r_ mesophase, we developed an additional generic interaction responsible for molecular registration. Thus, we have now accomplished a method that can generate (at least) three mesophases, biaxial nematic and two sanidics, that span almost the entire order–disorder scale between crystalline and amorphous states. This is achieved in a modular way, simply by activating terms in a series defining a phenomenological nonbonded potential.

We compared the structure of the Σ_r_ mesophase in our simulations with structures that have been experimentally determined^3,8^ for two highly ordered sanidic mesophases. The experimental studies established the structures of these mesophases on the basis of scattering data. Therefore, we discussed similarities and differences between the structures of sanidic mesophases in our simulations and the two experiments using their scattering patterns as a framework. The general hallmarks of sanidic Σ_r_ order are present in all three cases. However, there are differences in the way polymers register along the backbone axis. In the two experiments, backbone registration within each separate lamella occurred either only on the level of monomers^3^ or only on the level of entire chains^8^ (leading to smectic-like order within each lamella). The experiments reported no evidence that the order along the direction of polymer backbone is correlated across neighboring lamellae. In our simulations, in contrast, backbones register within each stack on both a monomer and chain level (manifested by a local lattice of monomers and smectic A intralamellar packing of polymers respectively). Moreover, we find a regular (smectic C) coupling of chain registration between different lamellae. Therefore, the Σ_r_ mesophase generated by our model is more ordered and closer to crystalline in structure than the mesophases reported in the two aforementioned experiments.

The model developed here is interesting for two reasons. First, sanidic materials are themselves useful for technological applications, in particular in organic electronics, where they are considered as processing intermediates for manufacturing solid state morphologies with favorable electronic properties.^4−6,12^ Second, our highly ordered Σ_r_ mesophase offers an approximation to crystalline states of board-like polymers that can be simulated at experimentally relevant length scales. In this work, we did not apply our model to any questions related to crystallization phenomena, but instead outline a few representative research directions for future studies.

One might wonder whether models with soft potentials, which by construction violate kinetic constraints imposed by noncrossability of chains in real materials, are at all applicable to polymer crystals, which are largely controlled by kinetic effects.^69−72^ We believe, however, that modeling the equilibrium structure of Σ_r_ mesophases while varying molecular features, such as chain topology, polydispersity, or deviations from molecular planarity^73^ (twisting), can help to answer certain questions. These include understanding the effects of altering these features on the general tendency of materials to crystallize, and the likelihood of forming conformational defects^74^ within crystallites.

Our model can be expanded to multicomponent polymers, where block copolymers with crystallizing blocks are of particular interest.^71,72,75^ Combining the expanded model with pseudodynamical Monte Carlo schemes that account for dynamic asymmetries between components^76^ would allow qualitative investigation into the interplay between microphase separation and crystallization. It is even worth applying such pseudodynamical stochastic algorithms to homopolymer melts. In this case, it is interesting to explore whether some mesoscopic features of the developing Σ_r_ grains, such as grain size, grain shape, chain folding, and bridging, bear similarities to actual semicrystalline materials.

## Supporting Information Available

Estimations of average density ρ_0_ and packing fraction Φ for various real polymers; further evidence of the quasi-long-range-order of stacking; demonstration of the origin of the monomer-level intralamellar lattice; insights into the origins of smectic order in our simulations; calculations for the descriptors of order used to create the phase diagram; order-of-magnitude analytical estimations for the values of λ and ζ required to promote biaxial nematic *N*_b_ and disordered sanidic mesophases Σ_d_ (PDF)
