# Supplementary material for: Mesoscopic Modeling of a Highly-Ordered Sanidic Polymer Mesophase and Comparison With Experimental Data
Source: J Phys Chem B. 2022 Mar 15;126(11):2285–98. doi: 10.1021/acs.jpcb.1c10599 (PMC8958507; doi:10.1021/acs.jpcb.1c10599)
Supplement: Supplementary file 1 — jp1c10599_si_001.pdf [file jp1c10599_si_001.pdf]

Supporting Information for: Mesoscopic Modeling of a Highly-Ordered Sanidic Polymer Mesophase and Comparison With Experimental Data

Emma L. Wood,[a] Cristina Greco,[a] Dimitri A. Ivanov,[b,c,d,e] Kurt Kremer,[a] and Kostas Ch. Daoulas*[a]

[a] *Max Planck Institute for Polymer Research, Ackermannweg 10, 55128 Mainz, Germany*

[b] *Institute for Problems of Chemical Physics, Russian Academy of Sciences, Semenov Prospect 1, 142432 Chernogolovka, Russian Federation*

[c] *Lomonosov Moscow State University, Leninskie Gory 1, 119991 Moscow, Russian Federation*

[d] *Institut de Sciences des Matériaux de Mulhouse, CNRS UMR 7361, 15 Jean Starcky, F-68057 Mulhouse, France*

[e] *Sirius University of Science and Technology, 1 Olympic Ave, 354340 Sochi, Russian Federation*

E-mail: daoulas@mpip-mainz.mpg.de

1 Calculation of Packing Fractions and Coarse-Grained Densities

Here, we estimate packing fractions Φ and density ρ_0 for various real polymers using our drastically coarse-grained (CG) framework.

Assuming that one CG monomer represents one actual monomer, the packing fraction of a real polymer is estimated from:

$$\Phi = \frac{\rho N_A}{M} \frac{4\pi L^3}{3} \tag{1}$$

Here ρ is the density of the material in $\mathrm{g\,cm^{-3}}$, M is the molar mass of monomers in

$g\,mol^{-1}$, N_A is the Avogadro constant, and L is a length that characterizes the size of each monomer in cm. It is reasonable to expect that monomers start interacting with each other when their side-chains come into contact, so we choose L to be the extended length of a side-chain in the all-trans state. For simplicity, for all polymers, we use for the density an approximate value $\rho = 1\,g\,cm^{-3}$.

We first consider poly(3-hexylthiophene) (P3HT), whose monomers have a molar mass of $M = 166\,g\,mol^{-1}$. The hexyl side-chains each consist of six C–C bonds, with bond lengths of 1.54 Å and bond angles of 112°,[1] so $L = 7.66$ Å $= 7.66 \times 10^{-8}$ cm. Substituting these values into eq 1 gives $\Phi = 6.8$.

Poly(3-dodecylthiophene) (P3DDT) is another polymer in the same family as P3HT, so we can calculate its Φ using the same values for bond length and angles. Monomers of P3DDT have a molar mass of $M = 250\,g\,mol^{-1}$ and the dodecyl side-chains each contain twelve C–C bonds, so $L = 15.3$ Å $= 1.53 \times 10^{-7}$ cm. Therefore, $\Phi = 36$.

We also estimate packing fractions for two other real polymers, PE12 polyester[2] and Ac-Ndc$_9$-Nte$_9$ polypeptoid,[3] because their scattering patterns are used later in the paper for qualitative comparison with our generic modeled mesophase. These polymers have more complex backbones than P3HT and P3DDT, and would therefore require an adapted CG model to simulate accurately, but we can still make a rough estimate of Φ.

For PE12 (chemical structure shown in Ref. 2), we treat the entire repeat unit, with molar mass of $M = 977\,g\,mol^{-1}$ as one monomer. Here, each side-chain consists of twelve C–C bonds of length 1.54 Å and two C–O bonds of length 1.43 Å. Assuming bond angles of 112° gives $L = 17.7$ Å $= 1.77 \times 10^{-7}$ cm, meaning that $\Phi = 14$. Note that the fact that there are four side-chains per monomer in PE12 rather than one in P3HT and P3DDT does not affect the packing fraction under our framework.

Ac-Ndc$_9$-Nte$_9$ polypeptoid (structure shown in Ref. 3) is a diblock copolymer constructed from two different monomers. However, as there are the same number of each type of monomer per chain, and the molar masses ($197\,g\,mol^{-1}$ and $217\,g\,mol^{-1}$) and extended side-chain lengths (12.8 Å and 13.5×10^{-9} Å) are similar, it is reasonable to use average values. This choice of $M = 207\,g\,mol^{-1}$ and $L = 13.1$ Å $= 1.31 \times 10^{-7}$ cm gives $\Phi = 28$.

We remind the reader that ρ_0 is defined as the number of monomers per σ^3. Taking

into account that $\sigma = L$ in our model, ρ_0 is estimated from:

$$\rho_0 = \frac{\rho N_A}{M}\sigma^3 = \frac{\rho N_A}{M}L^3 \quad (2)$$

Substituting the values presented during the calculations of Φ into eq 2, we obtain for P3HT, P3DDT, PE12, and Ac-Ndc$_9$-Nte$_9$ polypeptoid, ρ_0 = 1.6, 8.6, 3.4, and 6.5 monomers/σ^3 respectively.

2 Quasi-Long-Range Order of Stacking

In Figure S1 we present 1D correlation functions $g(r_z)$ for three of our Σ_r mesophases, equilibrated in simulation boxes of different sizes and shapes:

- Black line: $\{L_x, L_y, L_z\} = \{25.10, 21.36, 20.98\}$ with $\{n_x, n_y, n_z\}$ ={3,12,40}.
- Red line: $\{L_x, L_y, L_z\} = \{16.74, 10.68, 41.96\}$ with $\{n_x, n_y, n_z\}$ ={2,6,80}.
- Green line: $\{L_x, L_y, L_z\} = \{16.74, 7.12, 62.94\}$ with $\{n_x, n_y, n_z\}$ ={2,4,120}.

It is clear that as the box length L_z along the stacking direction z (and equivalently the number of stacking layers n_z) increases, the rate of decay of the peak heights in $g(r_z)$ also increases. This dependence of correlations on system size provides further confirmation[4] that our mesophase only has quasi-long-range order (QLRO) along the stacking direction.

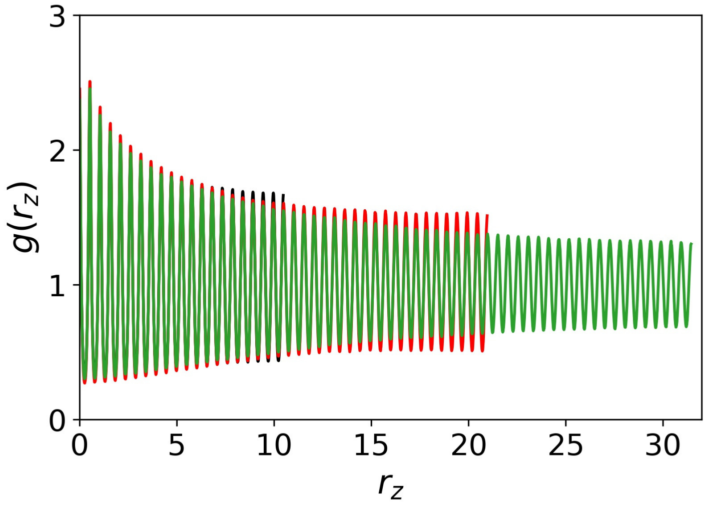

Figure S1 Comparison of $g(r_z)$ in systems with different box dimensions and different numbers of stacking layers n_z. Black: $n_z = 40$, red: $n_z = 80$, green: $n_z = 120$.

3 Origin of Intralamellar Monomer-Level Lattice

In this section, we demonstrate that the intralamellar monomer-level lattice (Figure 6a of the main paper) has a strong energetic driver. We present in Figure S2a the energy E_{nb} from all nonbonded interactions between monomers in a pair of polymers with $N = 16$, $\kappa = 0.874$, $\lambda = 0.408$, $\zeta = 0.204$ and $\eta = 1$. Figure S2b explains the geometrical setup of the polymers. Both chains are in the all-trans conformation (with $\theta = 150°$) and their orientation is fixed such that the backbones point along the x-axis and the stacking direction is along the z-axis. With the position of the first chain fixed, the second chain is translated in space. Figure S2a presents the calculated energy E_{nb} as a function of the coordinates r_x and r_z of the vector connecting the end monomers of the two chains, with $r_y = 0$ (see Figure S2b). The energy "surface" in Figure S2a changes very little on reversing the zig-zag direction of one of the oligomers.

Figure S2 demonstrates that the interference of attractive spherical shells of V_{reg} potentials between different monomers has two consequences. Firstly, it forms a low-energy band along the x-direction; the location of this band is consistent with the period of regular stacking along the z-direction (magenta line). Secondly, it creates a sequence of minima along the band, placed at $r_x = \pm 0.25, \pm 0.5, \ldots$. The deepest minima are found at $r_x = \pm 0.25$, which causes the system to maintain an x-shift of ± 0.25 between chains and produces an intralamellar lattice of monomer positions. In Figure S2c we provide a line graph showing the structure of the minima.

4 Origins of Smectic A and Smectic C Order

In our simulations, we find that SmA order is strongly preferred within each lamella. It is likely that this behavior has a strong energetic contribution, because the energy landscape favors close registration of chain centers of mass (see Figure S2c). However, the backbone shifts between centers of mass of polymers in successive stacking layers may be either $r_x = -0.25$ or $r_x = 0.25$, and there is no evidence that energetic contributions would discourage an overall displacement towards either positive or negative r_x via a random walk. However, it appears that such random displacements are suppressed, because intralamellar SmA order forms very fast in our simulations and remains strong during and after equilibration. Therefore, it is possible that there are also entropic contributions

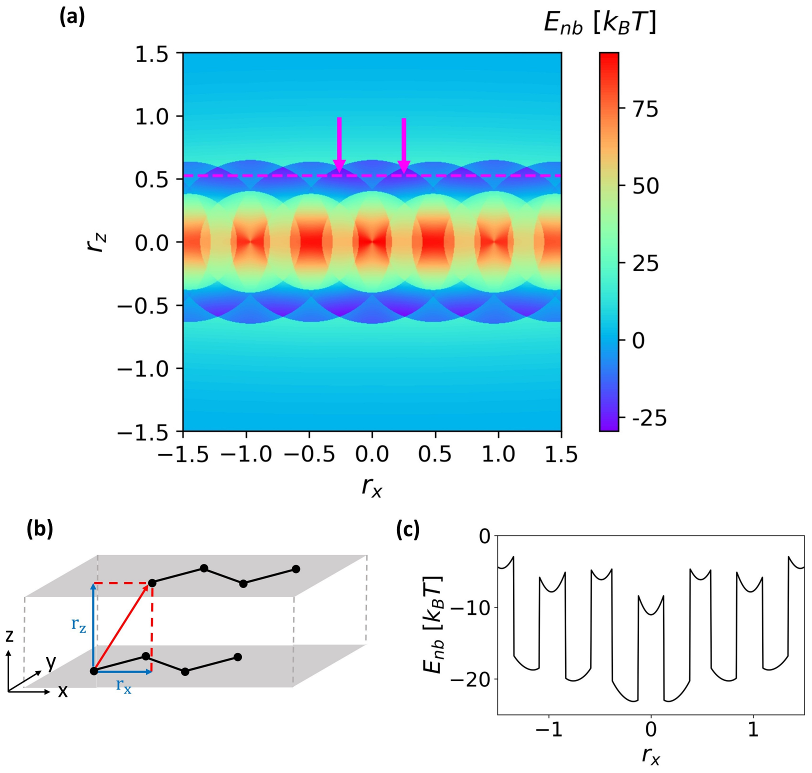

Figure S2 (a) Energy E_{nb} from all nonbonded interactions between the monomers of two $N = 16$ polymers in the all-trans conformation, with $\kappa = 0.874$, $\lambda = 0.408$, $\zeta = 0.204$ and $\eta = 1$. The geometric setup used in the calculation is illustrated in (b), where chains with only four monomers are shown for clarity (see text above for detailed description). The potential surface in (a) is shown as a function of the coordinates r_x and r_z of the vector connecting the end monomers of the two chains, while $r_y = 0$. The stacking distance is shown as a dashed magenta line, and arrows mark the minimum energy regions along this line. (c) Potential along r_x at the height of the stacking distance, i.e. along the magenta line, where $R_2 = 0.65$.

to SmA order, e.g. to increase the configurational entropy of the chain ends, or to give the orientation of each individual stack freedom to fluctuate slightly within the plane of its lamella.[5]

We also observe a SmC shift of chain backbones between polymers in neighboring lamellae. It is difficult to identify the reason for this behavior, because interlamellar monomer correlations are very weak (see Figure 6b in the main paper). To check whether the SmC state is energetically favored, we compare interaction energies in the SmC phase with interaction energies in systems purposefully prepared to have lamellae (i) in a SmA arrangement and (ii) randomly shifted with respect to each other.

These test systems are assembled by taking equilibrated Σ_{r} configurations and manually shifting each lamella individually along the x-direction to form the chosen struc-

tures. The configurations are locally equilibrated using the flip move (see Section 2.3 of the main paper) to relax monomer correlations without affecting the overall symmetries of the mesophases. For each of the two types of test systems, we set up 16 independent simulations, with $\{L_x, L_y, L_z\} = \{25.10, 21.36, 20.98\}$ and $\{n_x, n_y, n_z\} = \{3, 12, 40\}$ ($n = 1440$). As far as SmC phases are concerned, 32 samples (with the same dimensions as the test systems) are equilibrated. In half of them, the tilt angle of the SmC is 21.4° towards the left, whereas in the other half the same tilt angle occurs towards the right; these tilt angles are commensurate with the PBCs of the simulation box. Effectively, the samples with different tilt directions can be seen as repeats.

After equilibration, we calculate the total energy of each system, as well as the energies originating from intralamellar and interlamellar interactions separately. The mean values of these energies, for each type of symmetry, are plotted in Figure S3, where uncertainties denote the standard errors between repeats.

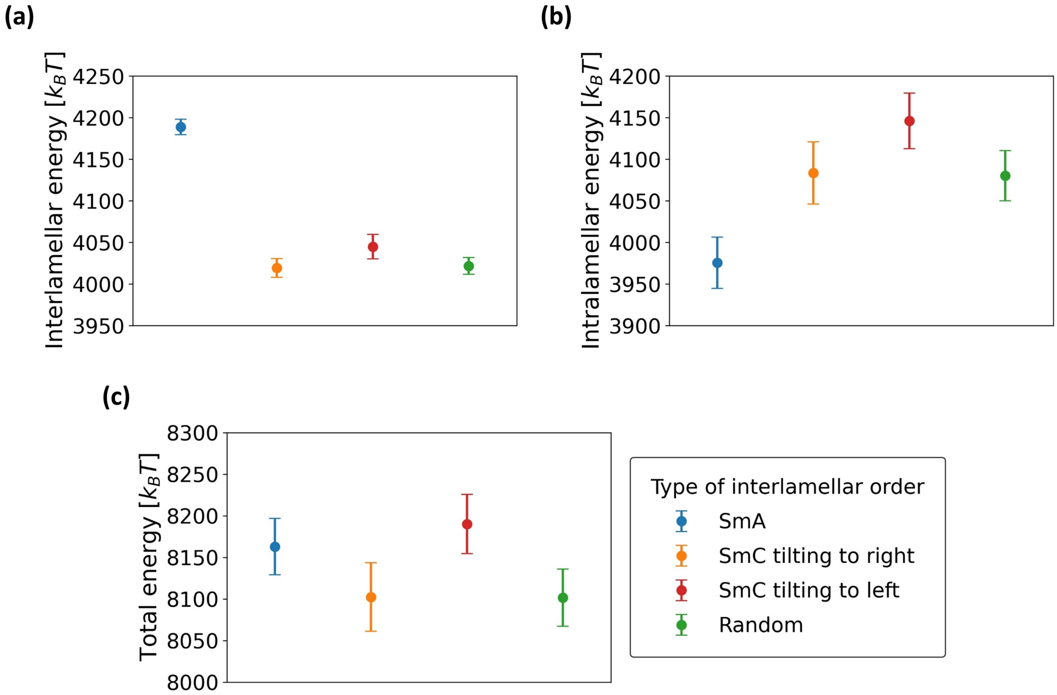

Figure S3 (a) Interlamellar, (b) intralamellar and (c) total energies for systems with SmA (blue), SmC (orange and red) and random (green) interlamellar order.

We find that the energies originating from interlamellar interactions are smaller for SmC and random arrangements than SmA ones. However, these energetic benefits appear to be compensated for by lower intralamellar interaction energies in the SmA systems, so that the total energies are similar for all symmetries. Therefore, the data shown in

Figure S3 (at least, for the accumulated statistics) do not provide clear indication that our SmC phase is driven by energetics. The SmC arrangement might offer entropic benefits, but their identification would require a free-energy based analysis, which is out of the scope of this study.

5 Descriptors of Order for Creating Phase Diagram

To construct the phase diagram shown in Figure 7 of the main paper, it is necessary to reliably determine which mesophase is exhibited by each of the systems studied. Although this can be judged to some extent by eye, various order parameters and phase descriptors are also used for quantification; these are discussed here.

5.1 Uniaxial and Biaxial Order Parameters, S and B

To distinguish biaxial nematics from isotropic melts, we use standard uniaxial and biaxial order parameters,[6] S and B. These are calculated for each system as follows; more details about the procedure can be found in Ref. 6.

Each monomer has an assigned set of orientation vectors $\boldsymbol{n}_j^{(1)}(s)$, $\boldsymbol{n}_j^{(2)}(s)$ and $\boldsymbol{n}_j^{(3)}(s)$, as described in Section 2.1 of the main paper. This allows us to define three tensors, Q^{11}, Q^{22} and Q^{33}, for each instantaneous configuration of the system:

$$Q^{kk} = \frac{1}{2}\left(\frac{3}{nN}\sum_{j=1}^{n}\sum_{s=1}^{N}\boldsymbol{n}_j^{(k)}(s)\otimes\boldsymbol{n}_j^{(k)}(s) - I\right) \tag{3}$$

Here, N is the chain length, n is the number of chains in the system and I is the identity matrix. $k = 1$, 2 or 3, depending on the orientation vector considered.

We then find the eigenvalues and eigenvectors for each of the matrices Q^{kk}. The dominant eigenvalue (i.e. the eigenvalue of Q^{11}, Q^{22} or Q^{33} with the highest absolute value) is defined as the uniaxial order parameter S, with its associated eigenvector providing the primary nematic director $\hat{\boldsymbol{n}}$ for the system. This direction is also labelled E_Z, with unit vector $\boldsymbol{Z}$, and the tensor corresponding to the dominant eigenvalue is labelled Q^{ZZ}.

We then consider the eigenvalue with the next largest positive value, and project its associated eigenvector onto the plane orthogonal to E_Z – this defines the secondary system director, labelled E_Y, with unit vector $\boldsymbol{Y}$. The corresponding tensor is labelled

Q^{YY}. The final axis E_X, with unit vector $\boldsymbol{X}$, is chosen to create a right-handed basis (i.e. $\boldsymbol{X} = \boldsymbol{Y} \times \boldsymbol{Z}$), with the remaining tensor labelled Q^{XX}. The biaxial order parameter B is then defined as follows, where the superscript T denotes taking the transpose of a vector:

$$B = \frac{1}{3}\left(\boldsymbol{X}^T Q^{XX} \boldsymbol{X} + \boldsymbol{Y}^T Q^{YY} \boldsymbol{Y} - \boldsymbol{X}^T Q^{YY} \boldsymbol{X} - \boldsymbol{Y}^T Q^{XX} \boldsymbol{Y}\right) \tag{4}$$

The order parameters S and B are then averaged over all relevant conformations.

5.2 Smectic A Order Parameter, Λ

A major difference between our Σ_d and Σ_r mesophases is the presence of SmA order within each lamella in the latter. We quantify intralamellar SmA order using a standard order parameter[7–9] Λ.

$$\Lambda = \left\langle \frac{1}{n_a} \left| \sum_{j=1}^{n_a} \exp\left(\frac{2\pi i [\boldsymbol{r}_{j,\mathrm{COM}} \cdot \hat{\boldsymbol{n}}_a]}{d_\mathrm{backbone}}\right) \right| \right\rangle \tag{5}$$

Here, n_a is the number of chains in the considered lamella a. $\boldsymbol{r}_{j,\mathrm{COM}}$ is the center of mass of each polymer j located in lamella a, and $\hat{\boldsymbol{n}}_a$ is the nematic director for the chain backbones in that lamella. d_backbone is the period of the SmA, estimated by dividing L_x (the box length in the backbone direction) by n_x (the number of chains lying along the x-axis in each stacking layer). Angular brackets indicate averaging over all lamellae in all conformations generated with the desired system parameters.

5.3 Cross-Lamellar Smectic C Order: Center of Mass Correlations

Cross-lamellar smectic order is generally quite easy to identify by visualization if the end monomers of each polymer are colored differently to the rest of the chain (e.g. Figure 4c of the main paper). However, this is only a qualitative analysis, so we also developed a quantitative method to test for the presence of cross-lamellar smectics. It is important to note that this method assumes well-formed intralamellar SmA layers.

We construct a correlation function between the centers of mass of polymers in neighboring lamellae:

$$b(r_x) = \left\langle \frac{L_x}{2n_a n_b} \sum_{j=1}^{n_a} \sum_{l=1}^{n_b} \delta\left(r_x - |r_{jl,\mathrm{COM};x}|\right) \right\rangle \tag{6}$$

When carrying out this calculation, we label our lamellae consecutively across the simulation box, and then consider each neighboring pair of lamellae (a and $b = a + 1$) in turn, taking into account periodic boundary conditions. n_a and n_b refer to the numbers of molecules in each lamella of the pair.

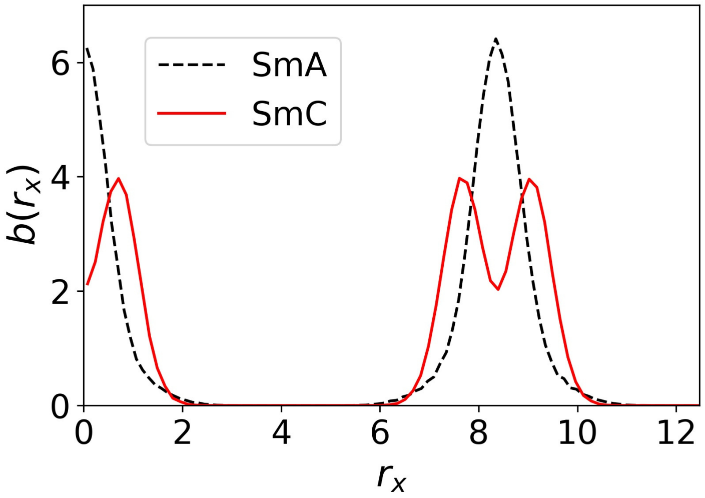

Figure S4 Comparison between smectic correlation functions for typical systems with SmA (black) and SmC (red) order, calculated using eq 6. In both cases, the chain spacing d_{backbone} along the backbone direction is 8.37. In SmA systems, maxima in $b(r_x)$ are found at the origin and at intervals corresponding to multiples of d_{backbone}. In SmC systems, characteristic double peaks are observed, with their maxima surrounding multiples of d_{backbone}.

We take two polymers j and l, one in each lamella a and b respectively, and project the center of mass of each onto the x-axis of the simulation box (i.e. the backbone direction). The modulus of the distance $r_{jl,\mathrm{COM};x}$ between the projections is placed into a histogram with bin size 0.25. This process is repeated for all possible pairs of monomers located across the chosen lamellae. We then average over all pairs of neighboring lamellae in all configurations, as indicated by the angular brackets.

Maxima in $b(r_x)$ at the origin and at intervals corresponding to multiples of the chain spacing d_{backbone} along the backbone direction signify SmA cross-lamellar order. Characteristic double peaks with the maxima surrounding multiples of d_{backbone} correspond to SmC (see Figure S4). The shift of the first maxima from the origin gives an indication of the SmC tilt angle. A similar correlation function considering only molecules in the same lamella can be used to confirm the presence of an intralamellar SmA.

6 Order-of-Magnitude Estimations for λ and ζ

We present analytical estimations to help understand which magnitudes of λ and ζ are needed to promote a biaxial nematic N_{b} and a disordered sanidic mesophase Σ_{d}, respectively. These estimations do not constitute a rigorous statistical mechanical theory, and are only back-of-the-envelope calculations which aim to provide a simple physical picture.

6.1 λ Parameter and Biaxial Nematic

We estimate the magnitudes of λ that are required to induce a transition from isotropic melt to biaxial nematic. All other anisotropic interactions are deactivated, that is to say we set $\zeta = 0$ and $\eta = 0$. We consider disconnected monomers, i.e. we neglect chain connectivity. The definitions of V_{biaxial} and the biaxial tensor in eq 5 and eq 8 of the main paper demonstrate that $\lambda V_{\mathrm{biaxial}}$ has a maximum value of $\lambda V_{\mathrm{biaxial(max)}} = \lambda U(r)$ when two board-like monomers, say s and m, are "orthogonal" to each other: $\mathbf{n}^{(1)}(s) \parallel \mathbf{n}^{(1)}(m)$, $\mathbf{n}^{(2)}(s) \perp \mathbf{n}^{(2)}(m)$ and $\mathbf{n}^{(3)}(s) \perp \mathbf{n}^{(3)}(m)$. The minimum value $\lambda V_{\mathrm{biaxial(min)}} = -\lambda U(r)$ corresponds to a perfectly biaxial orientation: $\mathbf{n}^{(1)}(s) \parallel \mathbf{n}^{(1)}(m)$, $\mathbf{n}^{(2)}(s) \parallel \mathbf{n}^{(2)}(m)$ and $\mathbf{n}^{(3)}(s) \parallel \mathbf{n}^{(3)}(m)$. Hence the maximum possible energy difference between pairs of monomers with different relative orientations is $\Delta(\lambda V_{\mathrm{biaxial}}) = 2\lambda U(r)$. We now substitute for r a length scale characterizing the structure of the liquid. Making the rough approximation that the local liquid packings in an isotropic melt and biaxial nematic are similar, we use the average distance between monomers $r_{\mathrm{av}} = 1/\sqrt[3]{\rho_0} = 1/\sqrt[3]{2.05}$ (in units of σ) to obtain $\Delta(\lambda V_{\mathrm{biaxial}}) = 2\lambda U(r_{\mathrm{av}}) \simeq 1.76\lambda$ (in units of thermal energy $k_B T$). We can expect that a biaxial nematic will appear, i.e. the symmetry of orientation will be broken, when $\Delta(\lambda V_{\mathrm{biaxial}})$ is on the order of $k_B T$. Hence, we take as a reference point $\lambda \simeq 0.57$, i.e. the value of λ where $\Delta(\lambda V_{\mathrm{biaxial}}) = 1$.

This reasoning involves several drastic simplifications. For example, it does not account for entropic effects and neglects cooperativity between monomers found in the same chain. Studies of uniaxial polymer nematics demonstrate[10–13] that chain stiffness correlates orientations of segments in the same molecule. These correlations enhance alignment forces between individual monomers and reduce the coupling strength that is required for an isotropic-nematic transition.

With these simplifications in mind, we compare our reference point with the phase

diagram in Figure 7 of the main paper. The simulations indeed demonstrate that $\lambda \leq 0.175$, which is equivalent to $\Delta(\lambda V_{\text{biaxial}}) \leq 0.3$, are indeed too weak to obtain a biaxial nematic melt. In Figure 7, a biaxial melt is observed at $\lambda = 0.291$, which corresponds to $\Delta(\lambda V_{\text{biaxial}}) \simeq 0.5$.

Furthermore, the requirement that $\beta V_{\text{lamella}} \geq 0$ (see main paper and Ref. 14) sets an upper boundary on λ. At each distance r, the most negative value of $\lambda V_{\text{biaxial}} + \zeta V_{\text{stack}} = -\lambda - 2\zeta$ occurs[14] when two interacting monomers are found in a perfectly biaxial, face-to-face registration. Hence, the condition $\beta V_{\text{lamella}} \geq 0$ leads to the constraint[14] $\kappa \geq \lambda + 2\zeta$. Therefore, when $\zeta = 0$, for our choice of $\kappa = 0.874$, it is necessary for $\lambda \leq 0.874$. When $\zeta > 0$, the region of allowed values of λ values shrinks further.

6.2 ζ Parameter and Disordered Sanidic Mesophase Σ_{d}

The stacking potential ζV_{stack} is introduced to act cooperatively[14] with the biaxial interaction $\lambda V_{\text{biaxial}}$, following the logic that stacking requires a parallel, i.e. biaxial, arrangement of planes of chain backbones. Therefore, we consider the emergence of a lamellar Σ_{d} mesophase as a transition from a state of biaxial nematic with uniform density ρ_0 into a state with biaxial order and density modulation. Our goal is to compare the energies of these two states as a function of ζ.

In our analytical calculation, both states contain an equal number of disconnected monomers in a volume V. Because spatial density modulation is affected by compressibility, we take into account the isotropic repulsion κV_{iso}, but set $\eta = 0$. Hence, the average energy $\mathcal{E}_{(\text{a})}$ per monomer has three contributions:

$$\mathcal{E}_{(a)} = \mathcal{E}_{\text{iso(a)}} + \mathcal{E}_{\text{biaxial(a)}} + \mathcal{E}_{\text{stack(a)}} \tag{7}$$

where $\mathcal{E}_{\text{iso(a)}}$, $\mathcal{E}_{\text{biaxial(a)}}$, and $\mathcal{E}_{\text{stack(a)}}$ are, respectively, the average energy (per monomer) due to the isotropic repulsive, biaxial, and stacking interactions. The subscript $\text{a} = \text{u}, \text{m}$ indicates the state: uniform or modulated density.

We first estimate the average energy $\mathcal{E}_{(\text{u})}$ in a biaxial nematic with uniform density distribution $\rho_{3\text{D}}(\mathbf{r}) = \rho_0$, where $\mathbf{r}$ is a vector in 3D space. The setup is illustrated in Figure S5a. We assume perfect biaxial alignment, such that for each monomer s, $\mathbf{n}^{(1)}(s) \parallel \hat{\mathbf{x}}$, $\mathbf{n}^{(2)}(s) \parallel \hat{\mathbf{y}}$ and $\mathbf{n}^{(3)}(s) \parallel \hat{\mathbf{z}}$, where $\{\hat{\mathbf{x}}, \hat{\mathbf{y}}, \hat{\mathbf{z}}\}$ are the unit vectors of the

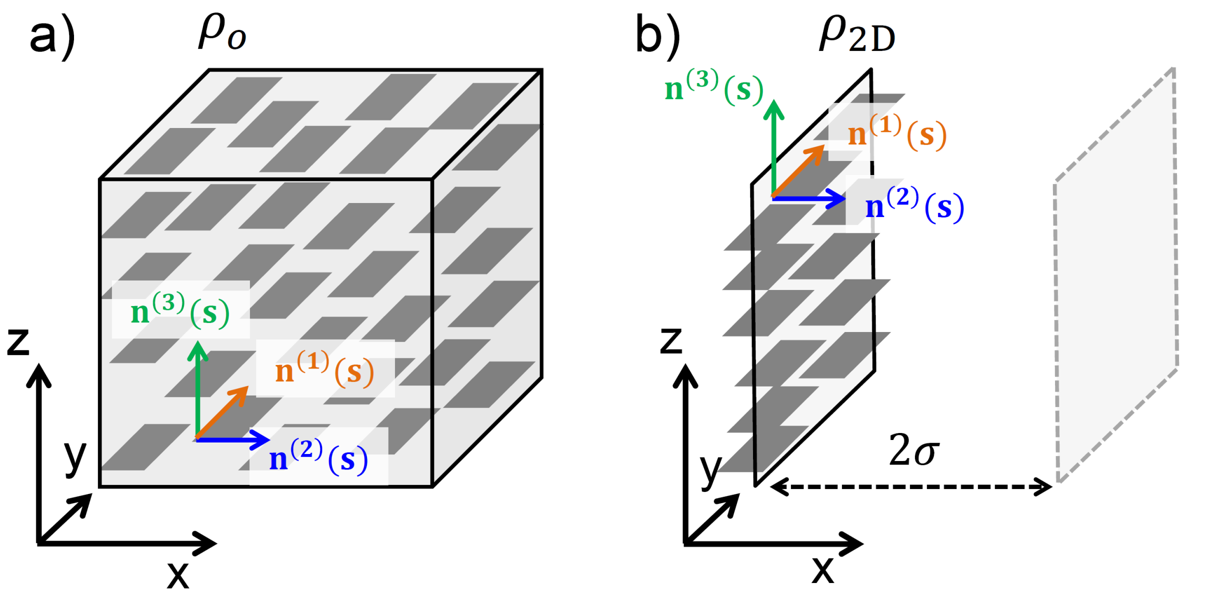

Figure S5 Cartoons clarifying the setups of the biaxial nematics with (a) uniform density and (b) modulated density used in the analytical calculations.

laboratory frame.

Within a mean-field ansatz, $\mathcal{E}_{\mathrm{iso(u)}}$ is given by:

$$\mathcal{E}_{\mathrm{iso(u)}} = \frac{\kappa}{2\rho_0 V} \int \int \rho_{3\mathrm{D}}(\mathbf{r}) U(|\mathbf{r}-\mathbf{r}'|) \rho_{3\mathrm{D}}(\mathbf{r}') d\mathbf{r} d\mathbf{r}' = \kappa \rho_0 2\pi \int_0^{2\sigma} U(r) r^2 dr = \frac{4}{3} \kappa \rho_0 \pi \sigma^3 \quad (8)$$

$\mathcal{E}_{\mathrm{biaxial(u)}}$ is estimated in a similar way, considering that, for perfect biaxial order, the biaxial potential between two monomers is $\lambda V_{\mathrm{biaxial(u)}} = -\lambda U(r)$ (see previous section):

$$\mathcal{E}_{\mathrm{biaxial(u)}} = -\lambda \rho_0 2\pi \int_0^{2\sigma} U(r) r^2 dr = -\frac{4}{3} \lambda \rho_0 \pi \sigma^3 \quad (9)$$

To calculate $\mathcal{E}_{\mathrm{stack(u)}}$ we consider that, for our specific orientation of the biaxial phase, the $V_{\mathrm{stack(u)}}$ between two monomers, say s and m, with distance vector $\mathbf{r}$ is:

$$V_{\mathrm{stack(u)}} = -U(r) \left[P_2 \left(\boldsymbol{n}^{(3)}(s) \cdot \hat{\boldsymbol{r}} \right) + P_2 \left(\boldsymbol{n}^{(3)}(m) \cdot \hat{\boldsymbol{r}} \right) \right] = -2U(r) P_2 (\hat{\mathbf{z}} \cdot \hat{\boldsymbol{r}}) \quad (10)$$

Therefore, using spherical polar coordinates:

$$\begin{aligned} \mathcal{E}_{\mathrm{stack(u)}} &= -\frac{\zeta}{\rho_0 V} \int \int \rho_{3\mathrm{D}}(\mathbf{r}) U(|\mathbf{r}-\mathbf{r}'|) P_2 (\hat{\mathbf{z}} \cdot (\mathbf{r}-\mathbf{r}')/|\mathbf{r}-\mathbf{r}'|) \rho_{3\mathrm{D}}(\mathbf{r}') d\mathbf{r} d\mathbf{r}' \\ &= -\zeta \rho_0 2\pi \int_0^{\pi} \int_0^{2\sigma} U(r) P_2(\cos\theta) r^2 \sin\theta dr d\theta = 0 \end{aligned} \quad (11)$$

Collecting all terms gives:

$$\mathcal{E}_{(\mathrm{u})} = \frac{4}{3}\rho_0\pi\sigma^3(\kappa - \lambda) \tag{12}$$

Next, we estimate the average energy $\mathcal{E}_{(\mathrm{m})}$ in a state with modulated density. For the modulation, we assume a simple profile, where the centers of mass of monomers are randomly distributed on infinitely thin parallel sheets (lamellae) with area A. The 2D density of monomers on each sheet is $\rho_{2\mathrm{D}}(\mathbf{r}) = \rho_{2\mathrm{D}}$, where $\mathbf{r}$ is a vector in 2D space. Furthermore, the distance between the sheets is 2σ; in other words, there are no interactions between monomers belonging to neighboring lamellae. The setup is illustrated in Figure S5b. Because the number of particles is conserved, $\rho_{2\mathrm{D}} = 2\sigma\rho_0$. Since lamellae do not interact with each other in this setup, we can calculate $\mathcal{E}_{(\mathrm{m})}$ by considering the energetics within one sheet only.

$\mathcal{E}_{\mathrm{iso(m)}}$ is given by:

$$\begin{aligned}\mathcal{E}_{\mathrm{iso(m)}} &= \frac{\kappa}{2A\rho_{2\mathrm{D}}}\int\int \rho_{2\mathrm{D}}(\mathbf{r})U(|\mathbf{r}-\mathbf{r}'|)\rho_{2\mathrm{D}}(\mathbf{r}')d\mathbf{r}d\mathbf{r}' \\ &= \kappa\rho_{2\mathrm{D}}\pi\int_0^{2\sigma} U(r)rdr = \frac{4}{5}\kappa\rho_{2\mathrm{D}}\pi\sigma^2 = \frac{8}{5}\kappa\rho_0\pi\sigma^3\end{aligned} \tag{13}$$

We estimate $\mathcal{E}_{\mathrm{biaxial(m)}}$, considering again that for perfect biaxial order, $\lambda V_{\mathrm{biaxial(m)}} = -\lambda U(r)$:

$$\begin{aligned}\mathcal{E}_{\mathrm{biaxial(m)}} &= -\frac{\lambda}{2A\rho_{2\mathrm{D}}}\int\int \rho_{2\mathrm{D}}(\mathbf{r})U(|\mathbf{r}-\mathbf{r}'|)\rho_{2\mathrm{D}}(\mathbf{r}')d\mathbf{r}d\mathbf{r}' \\ &= -\lambda\rho_{2\mathrm{D}}\pi\int_0^{2\sigma} U(r)rdr = -\frac{4}{5}\lambda\rho_{2\mathrm{D}}\pi\sigma^2 = -\frac{8}{5}\lambda\rho_0\pi\sigma^3\end{aligned} \tag{14}$$

To calculate $\mathcal{E}_{\mathrm{stack(m)}}$, we take advantage of eq 10 and use plane polar coordinates in the yz-plane, where ϕ is measured from the z-axis:

$$\begin{aligned}\mathcal{E}_{\mathrm{stack(m)}} &= -\frac{\zeta}{\rho_{2\mathrm{D}}A}\int\int \rho_{2\mathrm{D}}(\mathbf{r})U(|\mathbf{r}-\mathbf{r}'|)P_2\left(\hat{\mathbf{z}}\cdot(\mathbf{r}-\mathbf{r}')/|\mathbf{r}-\mathbf{r}'|\right)\rho_{2\mathrm{D}}(\mathbf{r}')d\mathbf{r}d\mathbf{r}' \\ &= -\zeta\rho_{2\mathrm{D}}\int_0^{2\pi}\int_0^{2\sigma} U(r)P_2(\cos\phi)rdrd\phi = -\frac{2}{5}\zeta\rho_{2\mathrm{D}}\pi\sigma^2 = -\frac{4}{5}\zeta\rho_0\pi\sigma^3\end{aligned} \tag{15}$$

Collecting all terms gives:

$$\mathcal{E}_{(\mathrm{m})} = \frac{4}{5}\rho_0\pi\sigma^3(2\kappa - 2\lambda - \zeta) \tag{16}$$

From eqs 12 and 16, we find the condition on ζ for which $\mathcal{E}_{(\mathrm{u})} > \mathcal{E}_{(\mathrm{m})}$, i.e. the modulated

state is energetically favorable:

$$\zeta > \frac{(\kappa - \lambda)}{3} \tag{17}$$

We now compare the condition in eq 17 with the phase diagram in Figure 7 of the main paper. For $\kappa = 0.874$ (and $\eta = 0$), eq 17 predicts that the Σ_d mesophase is preferable for $\zeta \geq 0.19$ when $\lambda = 0.291$, and $\zeta \geq 0.16$ when $\lambda = 0.408$. These predictions are close to $\zeta = 0.204$ and $\zeta = 0.175$, where the Σ_d is first observed in Figure 7 for the same values of λ. Of course, the agreement between the qualitative estimate and simulation data might be a fortunate cancellation of errors introduced by the approximations used to derive eq 17. These simplifications include neglecting polymer connectivity, entropic effects (such as differences in translational entropy between 3D and 2D molecular arrangements), and interactions across lamellae.

Analytically estimating the magnitude of η required for chain registration is even less straightforward than estimating ζ. For example, molecular architecture must be explicitly considered, because chain-chain registration is linked to the interference of non-bonded interactions between a monomer and several other monomers situated in a foreign chain, as demonstrated in Figure S2. Therefore, we do not provide an analytical estimation for η here.

References

[1] Mavrantzas, V. G.; Boone, T. D.; Zervopoulou, E.; Theodorou, D. N. End-Bridging Monte Carlo: A Fast Algorithm for Atomistic Simulation of Condensed Phases of Long Polymer Chains. *Macromolecules* **1999**, *32*, 5072–5096.

[2] Ebert, M.; Herrmann-Schönherr, O.; Wendorff, J. H.; Ringsdorf, H.; Tschirner, P. Sanidics: A New Class of Mesophases, Displayed by Highly Substituted Rigid-Rod Polyesters and Polyamides. *Liq. Cryst.* **1990**, *7*, 63–79.

[3] Greer, D. R.; Stolberg, M. A.; Xuan, S.; Jiang, X.; Balsara, N. P.; Zuckermann, R. N. Liquid-Crystalline Phase Behavior in Polypeptoid Diblock Copolymers. *Macromolecules* **2018**, *51*, 9519–9525.

[4] Frenkel, D.; Eppenga, R. Evidence for Algebraic Orientational Order in a Two-Dimensional Hard-Core Nematic. *Phys. Rev. A* **1985**, *31*, 1776–1787.

[5] Shakirov, T.; Paul, W. Crystallization in Melts of Short, Semiflexible Hard Polymer Chains: An Interplay of Entropies and Dimensions. *Phys. Rev. E* **2018**, *97*, 042501.

[6] Low, R. J. Measuring Order and Biaxiality. *Eur. J. Phys.* **2002**, *23*, 111–117.

[7] Polson, J. M.; Frenkel, D. First-Order Nematic-Smectic Phase Transition for Hard Spherocylinders in the Limit of Infinite Aspect Ratio. *Phys. Rev. E* **1997**, *56*, R6260–R6263.

[8] Mazza, M. G.; Greschek, M.; Valiullin, R.; Schoen, M. Role of Stringlike, Supramolecular Assemblies in Reentrant Supernematic Liquid Crystals. *Phys. Rev. E* **2011**, *83*, 051704.

[9] Maiti, P. K.; Lansac, Y.; Glaser, M. A.; Clark, N. A. Entropy-Stabilized Smectic C Phase in a System of Zigzag-Shaped Molecules. *Phys. Rev. Lett.* **2004**, *92*, 025501.

[10] Rusakov, V.; M. I. Shliomis, M. Landau-de Gennes Free Energy Expansion for Nematic Polymers. *J. Physique Lett.* **1985**, *46*, 935–943.

[11] Wang, X. J.; Warner, M. Theory of Nematic Backbone Polymer Phases and Conformations. *J. Phys. A: Math. Gen.* **1986**, *19*, 2215–2227.

[12] Ho, V.; Boudouris, B. W.; Segalman, R. A. Tuning Polythiophene Crystallization Through Systematic Side Chain Functionalization. *Macromolecules* **2010**, *43*, 7895–7899.

[13] Martin, J.; Davidson, E. C.; Greco, C.; Xu, W.; Bannock, J. H.; Agirre, A.; De Mello, J.; Segalman, R. A.; Stingelin, N.; Daoulas, K. C. Temperature-Dependence of Persistence Length Affects Phenomenological Descriptions of Aligning Interactions in Nematic Semiconducting Polymers. *Chem. Mater.* **2018**, *30*, 748–761.

[14] Greco, C.; Melnyk, A.; Kremer, K.; Andrienko, D.; Daoulas, K. C. Generic Model for Lamellar Self-Assembly in Conjugated Polymers: Linking Mesoscopic Morphology and Charge Transport in P3HT. *Macromolecules* **2019**, *52*, 968–981.
